# Dpath software reveals hierarchical haemato-endothelial lineages of Etv2 progenitors based on single-cell transcriptome analysis

**DOI:** 10.1038/ncomms14362

**Published:** 2017-02-09

**Authors:** Wuming Gong, Tara L. Rasmussen, Bhairab N. Singh, Naoko Koyano-Nakagawa, Wei Pan, Daniel J. Garry

**Affiliations:** 1 Lillehei Heart Institute, University of Minnesota, 2231 6th St S.E, 4-165 CCRB, Minneapolis, Minnesota 55114, USA; 2 Division of Biostatistics, School of Public Health, University of Minnesota, 420 Delaware St S.E., Mayo Building A302, Minneapolis, Minnesota 55455, USA

## Abstract

Developmental, stem cell and cancer biologists are interested in the molecular definition of cellular differentiation. Although single-cell RNA sequencing represents a transformational advance for global gene analyses, novel obstacles have emerged, including the computational management of dropout events, the reconstruction of biological pathways and the isolation of target cell populations. We develop an algorithm named *dpath* that applies the concept of metagene entropy and allows the ranking of cells based on their differentiation potential. We also develop self-organizing map (SOM) and random walk with restart (RWR) algorithms to separate the progenitors from the differentiated cells and reconstruct the lineage hierarchies in an unbiased manner. We test these algorithms using single cells from *Etv2-EYFP* transgenic mouse embryos and reveal specific molecular pathways that direct differentiation programmes involving the haemato-endothelial lineages. This software program quantitatively assesses the progenitor and committed states in single-cell RNA-seq data sets in a non-biased manner.

## Abstract (web summary)

Single-cell RNA sequencing has enabled great advances in understanding developmental biology but reconstructing cellular lineages from this data remains challenging. Here the authors develop an algorithm, *dpath*, which models the lineage relationships of underlying single cells based on single cell RNA seq data and apply it to study lineage progression of Etv2 expressing progenitors.

Cardiovascular lineages, including: blood, endothelium, endocardium, and myocardium, arise within a narrow time window from nascent mesoderm exiting the primitive streak and these lineages develop in synchrony to form the circulatory system. The haematopoietic and the endothelial lineages are closely related and express a number of common transcripts^1^. Based on the number of gene mutations that affect both haematopoietic and endothelial lineages, it has been proposed that that they arise from common progenitors^2^,^3^,^4^,^5^,^6^,^7^,^8^,^9^,^10^. The bifurcation point of these two lineages in embryos, however, has been debated and the gene expression profiles of the progenitors have not been fully defined, in part, due to the difficulty with the isolation of these bipotential cell populations.

Etv2, an ETS domain transcription factor, is critically required for endothelial, endocardial and haematopoietic development and has a negative impact on myocardial development^11^,^12^,^13^,^14^,^15^. Etv2 mutants are nonviable and completely lack haematopoietic and endothelial lineages. Furthermore, Etv2 overexpression in differentiating embryonic stem cells (ESs) induces the haematopoietic and endothelial lineages^13^,^16^. Etv2 is expressed in a narrow developmental window starting from embryonic day 7 (E7.0) and has diminished expression after E8.5 during murine embryogenesis^14^,^16^ Collectively, these data support a role for Etv2 in mesodermal differentiation at the junction of blood, endothelial and cardiac lineages. In the present study, we utilized Etv2-EYFP transgenic embryos^14^ and single-cell RNA-seq analysis to develop a blueprint of the lineage hierarchies of Etv2-positive cells early during development.

Single-cell RNA-seq provides an unprecedented opportunity to study the global transcriptional dynamics at the single-cell resolution^17^,^18^,^19^,^20^,^21^,^22^,^23^. Although multiple methods have been published to analyze the single-cell sequencing data, there are technical hurdles that need to be resolved in order to fully appreciate the biological impact. We developed mathematical solutions to two major issues encountered by the single-cell RNA-seq field. The first issue addresses the dropout events, arising from the systematic noise. This is a common problem in which an expressed gene observed in one cell cannot always be detected in another cell from the same population^24^. The presence of dropout events combined with sampling noise and the natural stochasticity and diversity of transcriptional regulation at the single-cell level^25^ makes profiling the low amounts of mRNA within individual cells extremely challenging. In the present study, we provide a weighted Poisson non-negative matrix factorization (wp-NMF) method as a solution to this problem.

The second outstanding issue is the need for additional biological information to determine the directionality of differentiation using the currently available methods. A number of conventional methods allow us to cluster cells into subpopulations and qualitatively associate the subpopulations with different cellular states during embryogenesis^19^. Recently, several single-cell RNA-seq analysis pipelines were developed to detect the branching trajectories and order single cells based on their maturity^23^,^26^,^27^,^28^. However, these methods required either a set of differentially expressed genes be predefined or the beginning and the end of the trajectory be determined by the investigator, limiting their general and non-biased applicability to a heterogeneous novel cell population. Here we develop a concept termed metagene entropy, which is combined with a self-organizing map (SOM) and random walk with restart (RWR) algorithms to separate the progenitors from the differentiated cells and reconstruct the lineage hierarchies in an unbiased fashion.

In these studies, we report solutions to these two major issues in the analysis of single-cell RNA-seq data. We develop an R package named *dpath* that decomposes the expression profiles with the awareness of the dropout events, quantitatively assesses the cellular state and prioritizes genes for both progenitor and committed cellular states. Importantly, we undertake a head-to-head comparison with commonly used factorization methods and pseudotime inference algorithms and demonstrate the superiority of the *dpath* program. Finally, we use *dpath* to decipher three major lineages of Etv2^+^ cells and identify key genes and signalling pathways for the group of progenitor cells with both endothelial and haematopoietic characteristics. This program, *dpath*, will facilitate and decipher the biological mechanisms that govern stem cell and progenitor cell populations.

## Results

### Single-cell RNA-seq analysis using the *dpath* pipeline

The *dpath* pipeline consists of four major steps. First, this program decomposes the expression profile matrix of single-cell RNA-seq into metagenes using wp-NMF. Second, *dpath* maps cells into metacells using a SOM algorithm. Third, the dpath algorithm prioritizes cells with respect to specific cellular states using a RWR algorithm on a heterogeneous metagene–metacell graph. Finally, this algorithm ranks genes for cellular states according to their expression profile (Supplementary Fig. 1a).

NMF is distinguished from principal component analysis (PCA) by its use of non-negativity constraints^29^. These constraints lead to a parts-based representation of subpopulations, instead of the holistic representations observed using PCA^29^. To account for the dropout events, we used a weighted Poisson model as the cost function for NMF. The expected gene expression level was modelled as the linear combination of non-negative metagene basis and coefficients. The observed gene expression level was then modelled as a mixture of Poisson distribution of expected expression level and a dropout event represented by a low-magnitude Poisson process^24^. When decomposing the single-cell expression profile, wp-NMF gave each entry a different weight depending on the odds of being a dropout event. The simulation study suggested, that in the presence of the dropout noise, wp-NMP was superior to PCA in the separation of the cell clusters on the low dimensional space and with regards to the *t*-distributed stochastic neighbour embedding of top principal components (Supplementary Fig. 1b)^30^.

**Figure 1:**
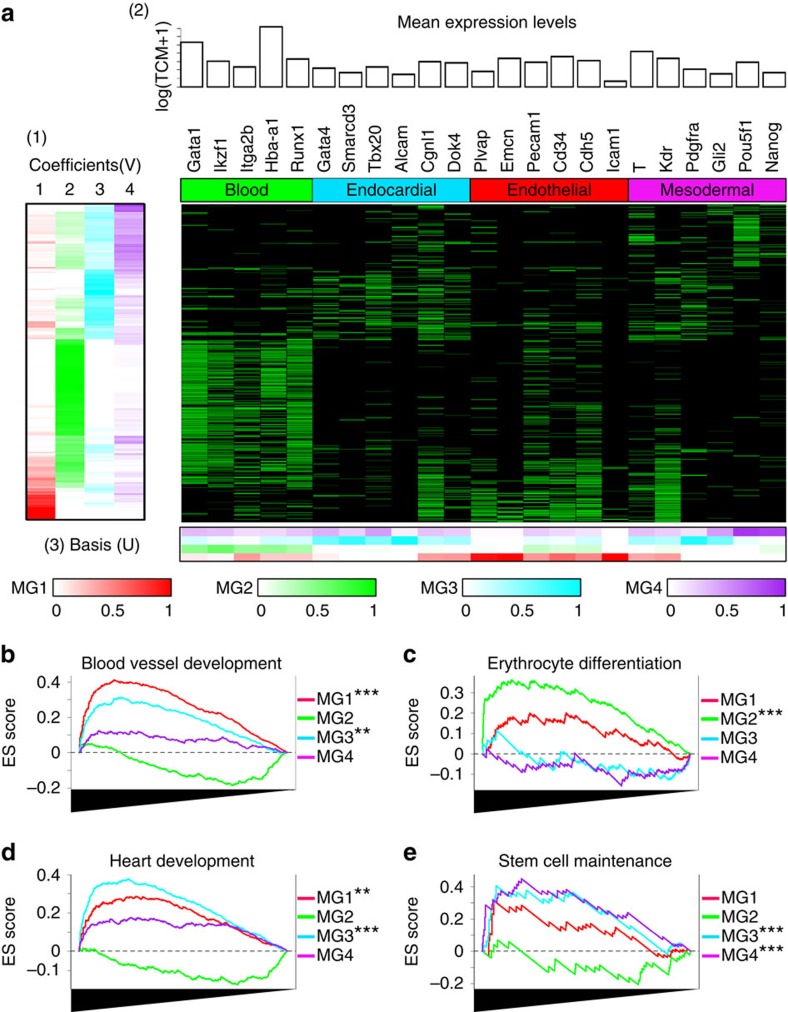
dpath successfully separated Etv2-EYFP^+^ cells into four metagenes. (**a**) Wp-NMF decomposed the expression profile matrix of Etv2-EYFP^+^ cells into metagene coefficients and metagene basis. Selected markers of expected lineages were used to identify the lineage represented by each metagene. (**1**) The heatmap showed the cell-wise metagene coefficients. Every row represented a single cell and the colour indicated the expression intensity of metagenes in each cell (cell-wise metagene signature). (**2**) Bar plot indicated the median expression levels of a list of known marker genes for haematopoietic, endocardial and endothelial lineages and the mesodermal progenitors across all 291 single cells. The heatmap showed each gene's observed cell-wise expression levels, scaled to a minimum of zero (black) and a maximum of one (green). (**3**) The heatmap showed the metagene basis for selected marker genes. Every column represented a gene and the colour indicated the contribution of each gene to each metagene. (**b**–**e**) GSEA showed that genes ranked by the correlation between their expression levels and cell-wise metagene coefficients of four metagenes were significantly associated with distinct Gene Ontology terms (*0.01≤*P* value <0.05; **0.001≤*P* value <0.01; ***P value <0.001. The statistical significance (nominal *P* value) was estimated by the permutation test. In each panel, *x* axis indicated the genes ordered according to the correlation between their expression levels and cell-wise metagene coefficients, and *y* axis indicated the ES score from the GSEA.

We used wp-NMF to decompose the expression profile matrix of 281 Etv2-EYFP^+^ cells captured from E7.25, E7.75 and E8.25 into four metagenes (Supplementary Fig. 1c,d)^31^. The expression matrix was therefore approximated by the product of non-negative metagene basis and coefficients. The metagene basis represented the contribution of each gene to each metagene, and the metagene coefficient, a probabilistic simplex that indicated the relative weight of each metagene in each cell, assigns distinct metagene signatures for individual cells (Fig. 1a).

To verify that this deconvolution strategy produced biologically relevant results, we first examined a list of selected genes with known expression patterns. The haematopoietic markers: Gata1, Ik2f1, Itga2b, Hba-a1, and Runx1, contributed to several metagenes, but primarily to the second metagene (MG2). The endocardial/cardiac genes: Gata4, Smarcd3, Tbx20, Alcam, and Dok4, contributed primarily to the third metagene (MG3)^32^,^33^,^34^. The mesodermal marker, Pdgfra, also contributed significantly to MG3, consistent with the previous observations that Pdgfra is expressed in the cardiac mesoderm^35^,^36^. Also the previously described endocardial marker, Cgnl1, contributed to MG1 and MG3 metagenes. The endothelial markers, Plasmalemma vesicle associated protein (Plvap), Endomucin (Emcn) and Icam1 contributed primarily to MG1. Interestingly, other common endothelial markers, such as Pecam1, Cd34 and Cdh5, contributed broadly to a number of metagenes. The broad contribution of several haematopoietic and endothelial markers supported the notion that the current lineage markers for these populations are not specific. In contrast, the mesodermal lineage marker Brachyury (T) and Gli2, a critical effector of sonic hedgehog signalling pathway, contributed strongly to MG4. Moreover, Pou5f1 and Nanog that are expressed at the primitive steak stage (E7.25) contributed exclusively to MG4 (refs 37, 38). The gene set enrichment analysis (GSEA) also suggested that genes that were specific to MG1 to MG4 were significantly associated with blood vessel development (GO:0001568), erythrocyte differentiation (GO:0030218), heart development (GO:0007507) and stem cell maintenance (GO:0035019), respectively (Fig. 1b–e). Collectively, these data demonstrated that four metagenes represented the endothelial, haematopoietic, endocardial lineages and mesodermal progenitors, respectively.

**Figure 2:**
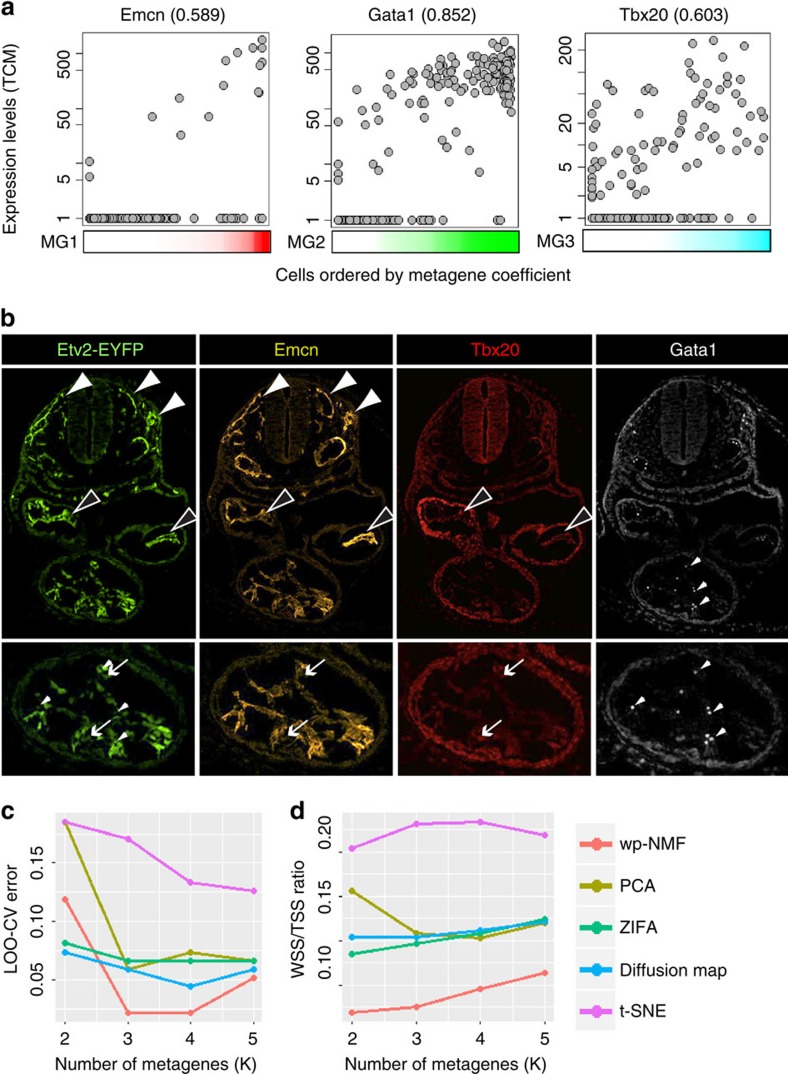
The metagene signature using wp-NMF successfully separated cell clusters with distinct spatial distribution. (**a**) The scatter plot showed the relationship between the expression levels of Emcn, Gata1 and Tbx20 and the metagene coefficients of MG1 (endothelium), MG2 (blood) and MG3 (endocardium). The Pearson's correlation coefficients in the parenthesis were computed between the expression levels and the metagene coefficients. (**b**) Immunohistochemical techniques were used to locate cell populations identified by the metagene signature. A transverse section (at the level of the heart) of an E8.25 mouse embryo was stained using antibodies to EYFP, Endomucin (Emcn), Tbx20 and Gata1 (from left to right). Note that EYFP-positive populations segregated into three distinct populations, EYFP^+^Emcn^+^Tbx20^−^Gata1^−^ endothelial cells (closed arrowhead), EYFP^+^Emcn^+^Tbx20^+^Gata1^−^ endocardial cells (open arrowheads) and EYFP^+^Emcn^−^Tbx20^−^Gata1^+^ blood (small arrowheads). (**c**,**d**) Wp-NMF had superior performance for the separation of Emcn^+^/Gata1^−^/Tbx20^−^, Emcn^−^/Gata1^+^/Tbx20^−^ and Emcn^−^/Gata1^−^/Tbx20^+^ among the Etv2-EYFP^+^ cells compared with PCA, dimensionality reduction for zero-inflated single-cell gene expression analysis, diffusion map and *t*-distributed stochastic neighbour embedding. In both panels, *x* axis indicated the number of hidden dimensions (*K*), and the *y* axis represented (**c**) leave-one-out cross validation (LOO-CV) error and (**d**) WSS (within-cluster sum of squares)/TSS (total sum of squares) ratio.

The observation that the single cells carrying different metagene signatures associated with different biological functions prompted us to hypothesize that the cells with a distinct metagene signature had a distinct spatial distribution. To experimentally test this hypothesis, we first identified Emcn, Gata1 and Tbx20 as the distinguishing marker genes for MG1 (endothelium), MG2 (blood) and MG3 (endocardium). Expression levels of these genes were strongly positively correlated with the metagene intensity of MG1, MG2 and MG3, respectively (Fig. 2a). Immunohistochemical staining demonstrated that the Etv2-EYFP^+^ cells segregate into three distinct subpopulations defined by these markers, namely, those exclusively marked by (1) Emcn (Etv2^+^/Emcn^+^/Gata1^−^/Tbx20^−^), (2) Gata1 (Etv2^+^/Emcn^−^/Gata1^+^/Tbx20^−^) and (3) Tbx20 (Etv2^+^/Emcn^−^/Gata1^−^/Tbx20^+^). These subgroups showed distinct spatial distribution in the E7.75 embryo (Fig. 2b) and confirmed that MG1 represented the endothelium, MG2 represented the blood and MG3 the endocardium. These results indicated that the metagene signature determined by wp-NMF was able to successfully separate three cell clusters with distinct spatial distribution. Moreover, wp-NMF had superior performance for the separation of these three spatially distinct Etv2-EYFP^+^ cell populations compared with other popular factorization and dimension reduction tools, such as PCA, dimensionality reduction for zero-inflated single-cell gene expression analysis, diffusion map and *t*-distributed stochastic neighbour embedding. To make these comparisons, we used the leave-one-out cross-validation (LOO-CV) and within-cluster sum of squares (WSS) to total sum of squares (TSS) ratio (Fig. 2c,d)^30^,^39^,^40^.

### Identification of progenitor and committed cells using dpath

**Figure 3:**
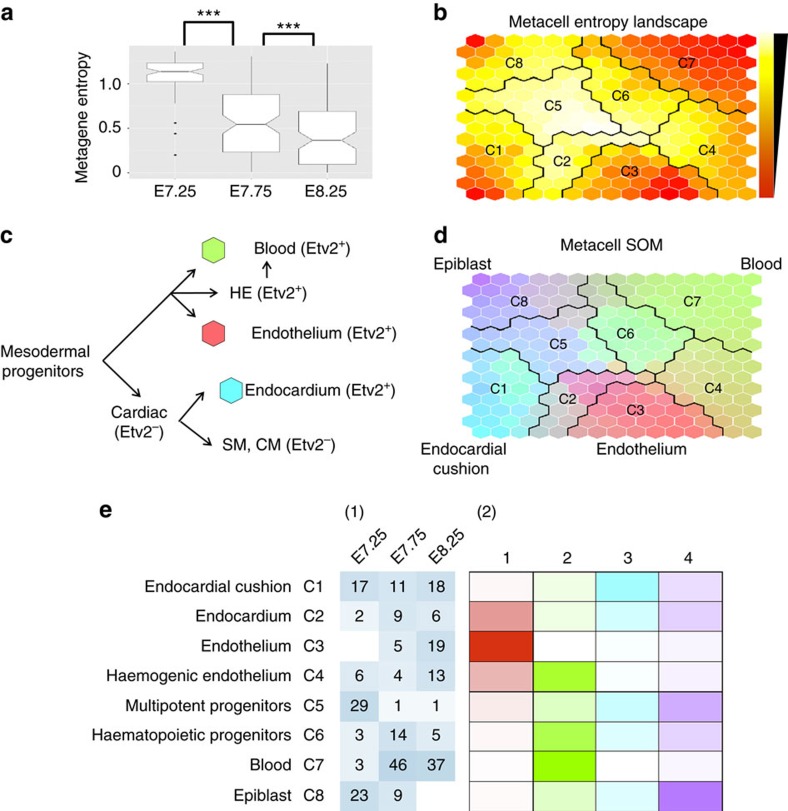
The metacell landscape and cluster analysis identified Etv2 derivatives. (**a**) The cells from E7.25 had significantly higher metagene entropy than the cells from E7.75, and the metagene entropy of E7.75 cells was significantly higher than E8.25 cells (Wilcoxon rank-sum test, *P* value=1.2E-10 and *P* value=0.00075). (**b**) The distribution of metagene entropy of metacells is shown on the SOM. White colour represents high entropy metacells and red colour represents low entropy metacells. (**c**) A schematic represents a simplified version of the expected differentiation pathway with the dominant metagenes represented by colour for populations we expected to observe. (**d**) PAM algorithm clusters metacells by partitioning the metacell. The colour indicates the expression intensity of each metagene in the metacells. (**e**) Eight major cell clusters were identified by partitioning the metacell landscape. Each cell was mapped to the metacell with the most similar metagene coefficients. (**1**) The table indicates the time sources of cells from each cluster. (**2**) The heatmap shows the average metagene coefficients of each cell cluster.

The metagene coefficient indicates the expression profile of each metagene in each cell. For example, MG1, MG2 and MG3 dominated isolated groups of cells (Fig. 1a). Alternatively, multiple metagenes could also be expressed in a single cell, suggesting that this cell harbored the gene signature of multiple lineages and is multipotent with regards to lineage commitment. Indeed, marker genes that are abundantly expressed in the committed lineages tend to be expressed in their common progenitor cells but at a lower level^19^,^41^. Thus we introduced the concept of metagene entropy as a novel tool to use the heterogeneity of gene expression signature of a single cell to predict the differentiation state of the cell^42^. Entropy is used in statistical mechanics and information theory as a measure of disorder or uncertainty. We hypothesized that cells with high metagene entropy have higher differentiation potential than cells with low metagene entropy. Our analysis using two published single-cell RNA-seq data sets on lung epithelium development and mouse fibroblasts reprogramming suggested metagene entropy was indeed significantly higher in progenitor cells compared with more differentiated cells (Supplementary Fig. 2)^19^,^43^. Following the application of metagene entropy to the Etv2^+^ cells, we noted that the cells from E7.25 had significantly higher metagene entropy than the cells from E7.75, and the metagene entropy of E7.75 cells was significantly higher than E8.25 cells (Wilcoxon rank-sum test, *P*-value=1.2E-10 and *P*-value=0.00075). This finding was consistent with the general consensus that cells from early developmental stages have higher differentiation potency than from the later stages (Fig. 3a). To our knowledge, this is the first method in this field of single-cell RNA-seq analysis that establishes a quantitative measurement of cellular (progenitor versus committed) state.

### Establishing the metacell landscape for Etv2 derivatives

Although we defined metagene entropy and established the directionality of the developmental programme, we introduced another requirement such that the metagene expression profiles between cells in the neighbouring developmental stages are similar. We used a SOM algorithm to organize Etv2^+^ cells with similar metagene coefficients into a hexagonal grid and visualized the lineage structures in a 15 × 15 two-dimensional (2D) map. The SOM is an unsupervised machine learning method that was developed to cluster and visualize the high dimensional data and has been widely used in bioinformatics because of its superb visualization capability^44^. In our application, each hexagonal grid on the SOM was defined as a *metacell*, and each cell was mapped to the metacell with the most similar metagene expression pattern. Our analysis revealed a graded distribution of metagene entropy on the SOM: in the central region of the SOM, the metacells had higher metagene entropy than those at the periphery or corners, and the region with the highest metagene entropy was enriched by the cells from E7.25. In contrast, the region with low metagene entropy was associated with more cells from the later developmental stages, E7.75 and E8.25 (Fig. 3b, Supplementary Fig. 3a–d). Moreover, the committed haematopoietic, endothelial and endocardial lineages were clearly separated or located at the edges and corners of the SOM (Fig. 3c,d). This metacell landscape therefore represented the lineage relationships reminiscent of the branching valleys of the epigenetic landscape envisioned by Waddington^45^ (Supplementary Fig. 3e).

Next, to reveal the identity of the major populations of Etv2-EYFP^+^ cells, we clustered all 281 cells into 8 cell clusters by partitioning the SOM using the Partitioning Around Medoids (PAM) algorithm (Fig. 3d,e). Among these cell clusters, C2, C4, C5 and C6 that expressed multiple metagenes were enriched in earlier time points (E7.25 and E7.75) and therefore, we hypothesized, represent the more progenitor-like cellular states. In contrast, C1, C3 and C7 expressed primarily the endocardial (MG3), endothelial (MG1) and haematopoietic (MG2) metagenes, respectively, and as such had relatively low average metagene entropy, supporting the notion that these cell clusters represent more differentiated cells (Fig. 3b and Supplementary Fig. 3f). C8 is a unique population that is found abundantly in E7.25 and disappears at later stages. As this population expresses pluripotency markers and very low level of Etv2 and EYFP (enhanced yellow fluorescent protein) compared with other populations, we hypothesize that these cells represent descendants of early blastomeres and epiblasts that express Etv2 at low levels or stochastically^46^.

We found that the metacells with the highest entropy in our cell population were positive for T (C5, highest expressors are marked with asterisks). This T^+^ group of cells clustered adjacent to the common haematopoietic and endothelial progenitors (highlighted in yellow) and represented the most immature progenitors present in our Etv2-EYFP population (Supplementary Fig. 4a). The metacells with highest entropy in C5 (demarcated by yellow lines) expressed Etv2, Kdr, Sox7, Runx1, Gata1 and Snca. Interestingly, these progenitors represented cells that expressed Sox7 and Runx1. The central location of these cells suggested that they were the earlier progenitors. In contrast, more mature cells of the haematopoietic and endothelial lineages segregated to peripheral locations. These peripherally located cells expressed Hbb-y, Car1 and Hba-a1, which are the mature markers of the haematopoietic lineage (C7), and Emcn, Plvap, and Nos3, which are the mature markers of the endothelial lineage (C3), respectively (Supplementary Fig. 6a).

Towards the lower left corner of the SOM, metacells enriched in endocardial/cardiac mesodermal genes (Tbx20 and Pdgfra) were localized. As these cells were isolated based on EYFP expression driven by the *Etv2* promoter, it is likely that C2 represents endocardium. To examine this hypothesis, we analyzed the expression of Cgnl1 and Dok4, which are reported to be enriched in endocardium^47^. We observed that both Cgnl1 and Dok4 were expressed in C2 population (Supplementary Fig. 4a,b). The segregation of the putative endocardium from the haematopoietic and endothelial lineages is consistent with previous reports that endocardium is derived from cardiac mesoderm (Fig. 3c)^48^.

### Biological verification of the dpath pipeline output

Our data demonstrated that, by combining the biologically relevant metagene signature, metagene entropy and the metacell landscape, the *dpath* pipeline provided a straightforward way to examine the lineage relationships of underlying single cells. Here we experimentally verified two predictions from analyzing the metacell landscape of Etv2-EYFP^+^ single cells.

**Figure 4:**
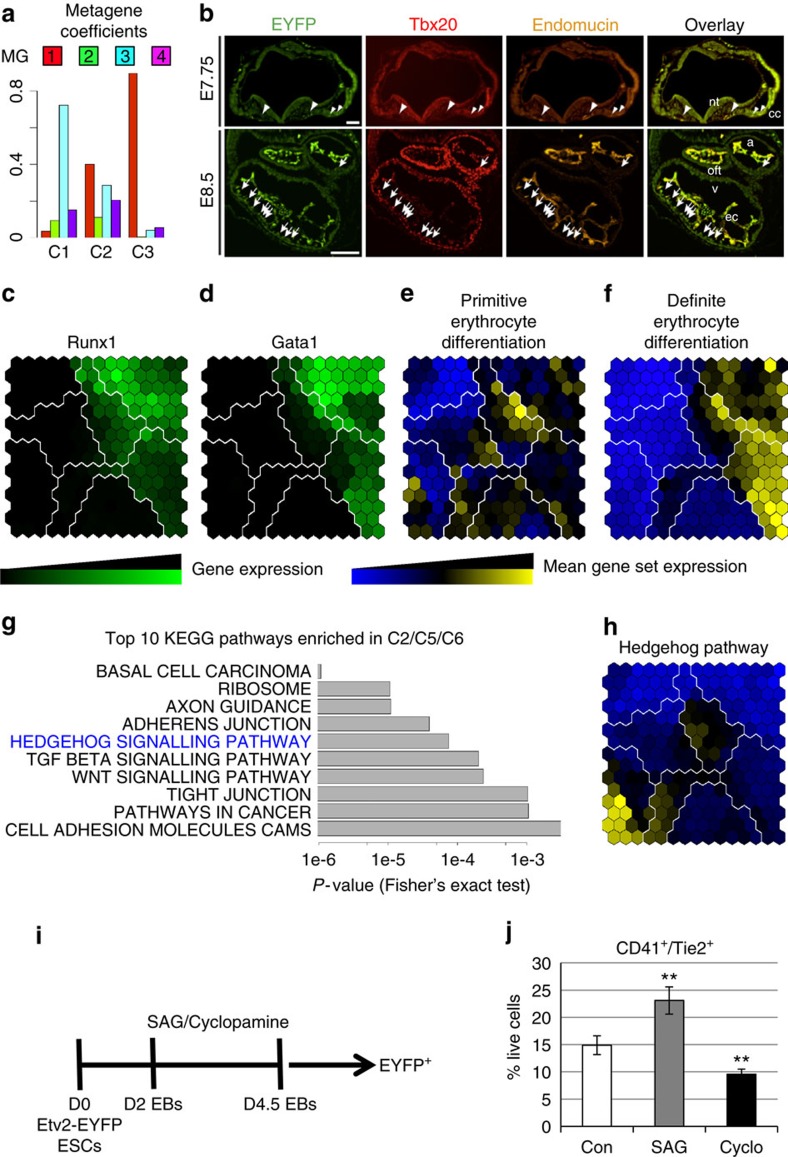
Immunohistochemical and ES/EB studies support the dpath results. (**a**) The average metagene coefficients of cells from the C1, C2 and C3 clusters were illustrated using the barplot. In contrast to C2 where both MG1 (endothelium) and MG3 (endocardium) had high intensity, C1 and C3 were dominated by MG3 and MG1, respectively. (**b**) Immunohistochemical analysis of Etv2-EYFP transgenic hearts at E7.75 and E8.5 supports the existence of the C2 cell population and the notion that they are progenitors of the cardiac cushion. Fluorescent images are pseudo-coloured after photographing in black and white. Large arrowheads point to EYFP^+^ Endomucin^+^ endothelial cells. Small arrowheads denote EYFP^+^ Endomucin^+^ angioblasts. Small arrows highlight EYFP^+^ Tbx20^+^ Endomucin^−^ C1 cells (a: common atrium, cc: cardiac crescent, ec: endocardium, ivs: intraventricular septum, la: left atrium, lv: left ventricle, nt: neural tube, oft: outflow tract, ra: right atrium, rv: left ventricle). Scale bars indicate 100 μm. (**c**,**d**) The expression patterns of Runx1 and Gata1 were illustrated on the metacell landscape. Green: high expression. Black: low expression. (**e**,**f**) The aggregated expression pattern of genes related to primitive erythrocyte differentiation (GO:0060215) and definitive erythrocyte differentiation (GO:0060216) were illustrated on the metacell landscape. Yellow: high expression. Blue: low expression. (**g**) The barplot shows the top 10 KEGG pathways that were enriched in genes that were significantly upregulated in C2, C5 and C6 cell clusters, compared with the remaining clusters (SCDE *P* value <0.001). (**h**) The aggregated expression pattern of genes related to hedgehog signalling pathway were illustrated on the metacell landscape. (**i**) A schematic diagram represents the ES/EB differentiation model system (using Etv2-EYFP transgenic cell lines) and the exposure to the SHH agonist (SAG) or SHH antagonist cyclopamine from days 2 to 4.5. (**j**) FACS quantification indicates that sonic hedgehog agonist (SAG) (or cyclopamine) significantly promotes (or suppresses) endothelial and haematopoietic progenitors (EYFP^+^/CD41^+^/Tie2^+^), compared with dimethyl sulfoxide control (*Student's *t*-test *P* value <0.05.

*Identification of endocardial cushion progenitors*. We first compared C1, C2 and C3 clusters, which were particularly intriguing. C2 had the metagene signature for endothelial, cardiac and mesodermal progenitors (MG1, MG3 and MG4), while C1 and C3 were dominated by the endocardial (MG3) and endothelial (MG1) metagenes, respectively (Fig. 4a). On the SOM, C2 connected C1 and C3 clusters and it had higher metagene entropy (Fig. 3b). We then predicted that the C2 population was the progenitors of the C1 population, according to their metagene coefficients and metagene entropy change. The gene profile analysis revealed that the general gene expression change was C2 (Etv2-EYFP^+^, Cardiac^+^, Endothelial^+^)→C1 (Etv2-EYFP^+^, Cardiac^+^, Endothelial^−^). This transition is similar to the endothelial–mesenchymal transition involved in the generation of cardiac cushion from the endocardium^49^. By using Emcn and Tbx20 as markers for MG1 and MG3, respectively, our immunohistochemical experiments confirmed the existence of the C2 cell populations and supported that the C2 population were progenitors of the cardiac cushion that originated from endocardium, and the molecular transition (that is, changes in gene expression profile) occurred as early as E8.25 (Fig. 4b, Supplementary Fig. 5 and Supplementary Note 1).

*Identification of two waves of haematopoiesis*. Next, we examined the paths leading to haematopoiesis. We observed that, on the SOM, between the highest entropy cell cluster C5 and the committed haematopoietic cluster C7, there existed a transitional cell cluster C6 with metagene entropy between C5 and C7, predicting that the differentiation path is C5→C6→C7. Within the C6 cluster, Runx1 was expressed in most of the C6 metacells, while Gata1 was only expressed in a few metacells near the border with C7 (Fig. 4c,d). This order of gene expression is consistent with the observation that Runx1expression preceeds Gata1 expression during primitive haematopoiesis^50^. C4 is another group that neighbours C7. The C4 cell cluster also had relatively higher metagene entropy than C7 and harbored the endothelial and haematopoietic metagenes; C4 had relatively stronger expression of genes related to definite erythrocyte differentiation (GO:0060216) than those related to primitive erythrocyte differentiation (GO:0060215), thus the C4 cell cluster represents the haemogenic endothelial lineage (Fig. 4e,f).

*Endothelial differentiation*. The C2 cell cluster was located between C5 and the committed endothelial cluster C3 and had an intermediate metagene entropy levels and served as a transition state between C5 and C3. Therefore, C5→C2→C6 transition represents the early differentiation of endothelial lineages.

*Identification of pathways for haematoendothelial bifurcation*. We hypothesized that the signalling pathways enriched in clusters C2, C5 and C6 have functional roles in the haemato-endothelial development. We identified 132 genes that were significantly upregulated in progenitor cellular clusters C2, C5 and C6, compared with the other five clusters (SCDE *P* value <0.001)^24^, and 21 KEGG pathways that were enriched in these upregulated genes (Fisher's exact test *P* value <0.05). Sonic signalling pathway (SHH) ranked as the fifth most enriched pathways in C2/C5/C6 (Fig. 4g,h). SHH has critical functions during development and regeneration^51^. To examine the roles of the SHH pathway in haemato-endothelial differentiation, we used an ES/embryonic body (EB) differentiation model system and exposed them to SHH agonist (SAG) or the SHH antagonist cyclopamine from days 2 to 4.5 (Fig. 4i). At day 4.5, we undertook FACS analysis for the Etv2-EYFP^+^ cells from respective ES/EBs and analyzed them for endothelial and haematopoietic markers. Compared with the dimethyl sulfoxide control, we observed that the SHH agonist significantly promoted the endothelial and haematopoietic progenitor cells (CD41^+^/Tie2^+^), while cyclopamine significantly suppressed this cell population (Fig. 4j). The SHH agonist and antagonist also had similar effects on differentiated endothelial (Tie2^+^/CD31^+^) and haematopoietic (CD41^+^/CD45^+^) lineages (Supplementary Fig. 6). These experiments confirmed SHH as a key signalling pathway that regulates the differentiation of haemato-endothelial lineages^52^,^53^,^54^,^55^. Our studies further established a new role for hedgehog signalling in the regulation of the haemato-endothelial (Etv2^+^Tie2^+^CD41^+^) progenitors (Fig. 4i,j).

### Discovery of trajectory from progenitor to committed state

**Figure 5:**
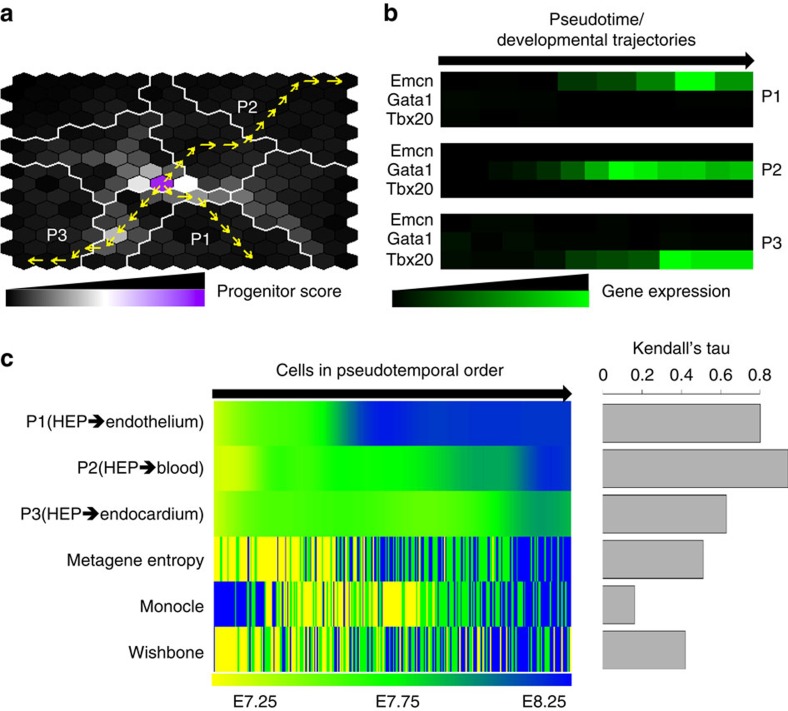
dpath allows the definition of the developmental trajectory and hierarchy of lineages. (**a**) The developmental trajectories were indicated from the high entropy progenitor (HEP) cellular state toward the committed cellular states of endothelium, blood and endocardium. The most likely progenitor cellular state and committed cellular states were determined by a RWR algorithms on a metagene–metacell heterogeneous graph. The developmental trajectories between the progenitor and committed cellular states were determined as the shortest paths (between the progenitor and the committed/differentiated cell) on the metacell landscape. P1, P2 and P3 represented the predicted developmental trajectories toward committed endothelial, committed haematopoietic and committed endocardial lineages. (**b**) The heatmaps show the expression pattern of Emcn, Gata1 and Tbx20 along the trajectories P1, P2 and P3. (**c**) The Kendall rank correlation coefficients between the pseudotime and temporal labels (E7.25, E7.75 and E8.25) were used to evaluate the performance of trajectory inference. For *dpath*, the lineage-specific cells were ordered into pseudotemporal order along three separate trajectories P1, P2 and P3, respectively. The cells were also reordered merely based on their metagene entropy. For Monocle and Wishbone, we used the cell-wise pseudotime reported by the algorithms.

After organizing cells into a SOM such that neighbouring metacells had a similar metagene expression pattern and establishing metagene entropy as a means to add directionality to the differentiation process, we next quantitatively assessed the progenitor and committed states on the metacell SOM and predicted the developmental trajectories. We first built a heterogeneous metacell–metagene probability graph (a transition matrix) to describe the probability of transitioning from a metagene to a metacell (or vice versa) or from a metacell to another metacell. A metacell was considered as a parent (progenitor) of its neighbouring metacell on the SOM only if its metagene entropy was higher than that of the derivative metacell. Second, we used a RWR algorithm on the heterogeneous graph to infer the probability of a metacell being in a committed state to one metagene or being in a progenitor state with the ability to transition to multiple metagenes^56^. Once the most likely progenitor and committed states (metacells) were identified, developmental trajectories from the progenitor cellular states toward the committed cellular states of endothelium, blood and endocardium were determined as the shortest paths between them on the SOM (Fig. 5a).

First, we verified the inferred the progenitor and committed cellular states. We ranked genes according to the similarity between their metacell expression profiles and the probability of being a specific cellular (progenitor or committed) state of the metagene(s) (Supplementary Fig. 7). The GSEA suggested that regulation of vasculature development (GO:1904018), erythrocyte differentiation (GO:0030218) and epithelial to mesenchymal transition (GO:0001837) related genes were significantly enriched at the top ranking genes for the committed metacells for the first, second and third metagenes, while the cell fate commitment (GO:0045165) related genes were significantly correlated with both MG3 and MG4, which was consistent with our previous determination of metagene fates based on known marker genes (Supplementary Fig. 8a–d)^57^. The GSEA of known mouse phenotypic-related genes suggested similar functional separations of metagenes (Supplementary Fig. 8e–g). To further confirm the biological relevance of genes that are enriched in predicted progenitor cellular states, we examined a previously published Etv2 chromatin immunoprecipitation sequence data set and found that the genes that had experimentally verified 3,953 highly confident (common in their V5 and PolyAb experiments) Etv2-binding sites (at least one chromatin immunoprecipitation sequence hit within −5,000 to +1,000 bp region surrounding the transcription start sites of at least one transcript) had significantly greater prioritization scores than those that did not (Wilcoxon rank-sum test, *P* value <1.0 × 10^−20^, Supplementary Fig. 8h)^58^. These results verified the biological relevance of progenitor and committed cellular states inferred by the RWR algorithm.

Second, by examining the expression of three known lineage marker genes (Emcn, Gata1 and Tbx20) along the *dpath*'s developmental trajectories, we found that Emcn, Gata1 and Tbx20 were upregulated along the endothelial path (P1), haematopoietic path (P2) and endocardial path (P3) (Fig. 5b). We then undertook a head-to-head comparison and evaluated whether currently available methods can predict the trajectories that we obtained. Results show that Monocle, Wishbone and Mpath were not able to infer the pseudotemporal or developmental trajectories that agree with the current biological knowledge (Supplementary Fig. 9). Moreover, to quantitatively evaluate the accuracy of inferred pseudotime, we counted how often a pseudotime puts a cell from a later temporal sorting before an earlier one (measured by Kendall rank correlation coefficient). We found that there existed a strong positive correlation between temporal labels (E7.25, E7.75 and E8.25) and *dpath*'s pseudotime (mean Kendall rank correlation coefficient=0.798), which was noticeably higher than popular pseudotime inference algorithms such as Monocle (0.213) and Wishbone (0.375) (Fig. 5c, Mpath was excluded from this comparison as the pseudotime could not be automatically calculated).

Taken together, the GSEA of genes that were enriched in committed and progenitor cellular states confirmed the biological significance of developmental trajectories predicted by *dpath*, and the results also suggested that the predicted pseudotime was more accurate than Monocle and Wishbone.

## Discussion

Here we describe the use of the *dpath* pipeline to decompose single-cell RNA-seq data with the awareness of dropout events. We provide three major technical breakthroughs to the single-cell analysis technology that includes: (1) a method to fill in dropout events; (2) a method to rank the differentiation potential using the metagene entropy, and (3) a method to visualize the differentiation paths on a 2D map. We used this method to prioritize committed and progenitor states for haematopoietic, endocardial and endothelial lineages obtained from 281 Etv2^+^ cells and ranked genes for distinct cellular states, especially for progenitor endothelial and haematopoietic states.

The first unique feature of *dpath* is applying wp-NMF for decomposing single-cell RNA-seq data. The use of the weighted Poisson model as the cost function reduced the impact of dropout events on matrix decomposition by maximizing the usage of informative genes that have a high probability of being expressed. The other advantage of NMF-based matrix decomposition method, compared with PCA, is that NMF yields a sparse parts-based representation of gene expression profiles^31^. Just as NMF is able to distinguish different meanings of words used in different contexts, metagene basis and coefficients can overlap and thus expose the participation of a single gene in multiple pathways and account for the activity of multiple pathways in a single cell. As a result of the parts-based representation, the metagene entropy, the entropy of metagene coefficients after proper scaling, serves as a measure of how many distinct programmes (parts) are active (expressed) in a cell. A cell with high metagene entropy implies that multiple programmes (represented by metagene basis) participate in the cellular activity, leading to a high uncertainty with respect to the lineage commitment and thus high level of cellular plasticity^59^. We applied *dpath* to publicly available single-cell data sets and undertook a head-to-head comparison with conventional programs. We demonstrated the superiority of *dpath* as it accurately predicted differentiation states and had higher resolution than previously published methods. Although entropy has been described as a potential measure for the uncertainty concerning the cellular state, to our knowledge, this is the first study to establish an entropy-based method to measure the multipotency in the context of single-cell expression analysis^42^.

Another unique feature of our new package *dpath* is that it represents the cellular states on a 2D SOM where metacells with similar metagene expression profiles are grouped together. This not only provides an intuitive way to visualize the distribution of cellular states from the input cells but also reduces the impact of dominant lineages in the analysis. Another important feature of this method is that one metacell is allowed to have multiple parental states, and globally, there can be multiple progenitor states that can give rise to individual committed states. This provides additional flexibility of modelling lineage hierarchies than organizing cells into a lineage tree-like structure where all individual committed states originate from one single cell, because single-cell transcriptome analysis represents a snapshot of cells present at experimental time points (E7.25, E7.75 or E8.25, in this case), and any given cell is unlikely to be a descendant of similar cells present at the same time. Therefore, SOM reflects continuous differentiation paths of multiple cells that are asynchronously differentiating towards the same differentiated state.

To further examine the dpath algorithm, we interrogated a subpopulation of the Etv2-expressing cells during murine embryogenesis. The high entropy progenitor cells of the haematopoietic and the endothelial lineages that we have identified are of intense interest, with respect to lineage specification. At E7.25 (early streak), E7.75 (late streak—late allantoic bud stage^60^) and E8.25 (linear heart loop stage), Etv2-EYFP^+^ cells are present in endothelial cells and primitive erythrocytes of the yolk sac blood islands (extraembryonic) and embryonic blood vessels, including dorsal aortae, endocardium and migrating angioblasts^14^. Moreover, previous studies are consistent with the notion that prior to gastrulation epiblast cells are largely unspecified, and the signals they encounter as they ingress through the primitive streak specifies their fate^60^,^61^. New mesodermal cells emerging from the streak are still plastic but commit quickly to specific lineages based on the signals they received in the primitive streak. Differential enrichment of multiple signalling pathways in haematopoietic and endothelial metacells indicate that these are candidates that cells encounter as they pass through the primitive streak. In the present study, we used *dpath* to successfully identify the dynamic expression pattern of the members of SHH signalling pathway and experimentally verify its critical roles in haemato-endothelial lineage differentiation. We do recognize that the number of profiled cells was relatively small compared with the total population of Etv2^+^ cells *in vivo*, especially for the later time point E8.25 (Supplementary Table 1). Although we have successfully identified the major developmental trajectories within the Etv2^+^ cells, addition of more single cells will reveal further subpopulations within committed endothelial, endocardial and haematopoietic lineages.

In summary, using the *dpath* pipeline, we successfully clustered single-cell RNA seq data without using previously known information, which was then verified by gene expression analysis and functional analysis. The expression patterns of known genes and calculated metagene entropy were consistent with known differentiation pathways of haematopoietic and endothelial cells. Our data are significant in multiple ways. First, we provide the full transcriptome of individual Etv2^+^ cells, which was not available previously. This is important as many genes are commonly expressed in haematopoietic and endothelial lineages. Cell surface markers commonly used to distinguish them from each other or their progenitors are not highly specific. Here we analyzed the transcriptome of single cells that provides information for identifying novel markers of these cell populations to improve the purity of populations for transcriptome and functional analyses. Second, we identified differentiation paths from progenitors to more mature cells using the novel concept of metagene entropy. The gene expression observations within the SOM differentiation paths validate the method and attests that this concept is an excellent approximation of the differentiation process. We predict that this method will be able to reconstruct differentiation pathways with any data set, including different populations and broader, heterogeneous data sets. Third, pathway enrichment analysis based on our results identified signalling pathways and molecules that were not previously identified as well as those that have been previously identified. Finally, we provide the *dpath* pipeline in a downloadable R package. This will be an essential tool to extract meaningful information from exponential amounts of RNA-seq data produced daily.

## Methods

### Cell isolation

Etv2-EYFP embryos were harvested from time mated females at E7.25, E7.75 or E8.25 and screened using microscopy for EYFP expression^14^. Embryos were divided into EYFP-positive and -negative pools for dissociation with TrypLE Express (Gibco by Life Technologies). After dissociation, cells were diluted with 10% foetal bovine serum in DMEM and pelleted at 1,000*g*. Cells were resuspended in 0.1% propidium iodide and 2% serum in PBS. EYFP-negative embryos were used as a gating control sample. Propidium iodide-negative, EYFP-positive cells were sorted by FACS using a MoFlo XDP (Beckman Coulter) into DMEM plus 10% foetal bovine serum. FACS sorted cells were resuspended at 500 cells μl^−1^ before loading onto a Fluidigm 10–17 μm integrated fluidics circuit for capture, viability screening, lysis and library amplification on a C1 Single-Cell Auto Prep System (Fluidigm).

### Single-cell RNA-seq

Libraries were analyzed for cDNA content by pico green staining. Wells that contained a single viable cell and at least 0.2 ng μl^−1^ cDNA were chosen to proceed with sequencing. All libraries were sequenced using 75-bp paired end sequencing on a MiSeq (Illuminia), generating 202K–1,910K paired end reads for each cell. The cells with <100K paired reads were removed, resulting in 281 cells for analysis. The transcripts per million (TPM) estimates were obtained with TopHat (v2.0.13) and Cufflinks (v2.2.1)^61^. The median mapping rate was 88.3%. Among 14,480 genes that could be detected in at least two cells (TPM≥1), we fitted a noise model with respect to each gene's mean and coefficient of variance (CV, s.d. divided by the mean) as 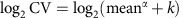. Then we removed 1,448 genes with high technical noise, which were furthest from the fitted line^62^,^63^. We also removed 7,240 ubiquitously expressed genes whose CV was below the median CV. The resulting 5,799 genes were used for the following analysis.

### Weighted Poisson non-negative matrix factorization

Let **X**_*nm*_ be the log-transformed TPM of gene *n* in cell *m*. We hypothesized that the expected log-transformed TPM of gene *n* in cell *m*, **μ**_*nm*_, could be represented as:


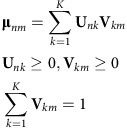


where *K* was the number of metagenes, **U**_*nk*_ was the metagene basis indicating the contribution of gene *n* on the *k*th metagene and **V**_*km*_ was the metagene coefficient indicating the expression profile of the *k*th metagene in cell *m*. Specifically, the expected gene expression level was modelled as the linear combination of non-negative metagene basis and coefficients. The cell-wise metagene coefficients were summed up to one.

Similar to work by Kharchenko *et al*.^24^ on the identification of differentially expressed genes in single-cell RNA-seq data, we defined a weighted log-likelihood function for an observed expression level of gene *n* in cell *m* as:


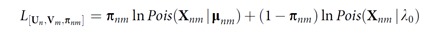


where **π**_*nm*_ ranges from zero to one, approximating the likelihood of gene *n* being expressed in cell *m*, that is, the probability that observed expression level **X**_*nm*_ follows a Poisson distribution with the mean as **μ**_*nm*_. The dropout event was also modelled as a Poisson distribution with the mean as *λ*_0_=0.1. As it was reasonable to hypothesize that **π**_*nm*_ was proportional to the probability of being expressed, it could be estimated by:


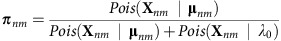


**π**_*nm*_ could be viewed as a weight for the observed expression level of gene *n* in cell *m*, depending on the probability of being expressed over that of a dropout event.

Taken together, to decompose expression matrix into metagene basis and coefficients, we solved such a constrained maximization problem:


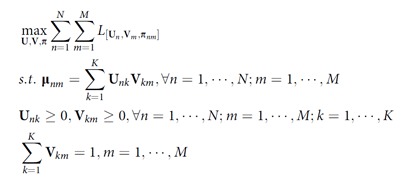


Similar to solving a regular NMF problem with cost functions as Euclidean distance or Kullback–Leibler divergence, we optimized the objective function using a gradient ascent method and multiplicative rules to iteratively update **U** and **V**, until convergence or maximum iterations were reached:


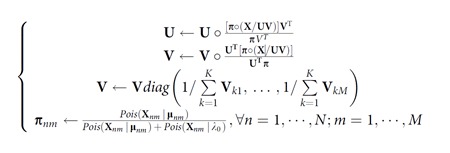


where ‘∘' was the Hadamard matrix product, ‘/' was the element-wise division and ‘*diag*(….)' represents a diagonal matrix where diagonal entries are indicated by ‘…'.

To accelerate the convergence, weighted NMF was used as a burn-in phase to initialize **U** and **V**, where a fixed weight of *w*_0_=0.1 was given to the zero entries in the gene expression matrix **X** and a weight of one to non-zero entries^64^. In weighted NMF, **V** was initialized using non-negative singular value decomposition^65^.

The metagene entropy of cell *m* was defined as 
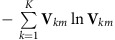
.

### Choice of the size of metagene *K*

As the objective function was not convex, wp-NMF may or may not converge to the same solution on each run, depending on the initialization of **U**. The wp-NMF was repeated for *r*_*mf*_ times with different random initialization of **U**. The consensus matrix 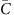 and the cophenetic correlation coefficients 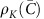
were computed as described in Brunet *et al*.^31^. We selected values of *K*=4 where the magnitude of the cophenetic correlation coefficient began to fall (Supplementary Fig. 3a). Our experiments also suggested that *r*_*mf*_=20 was sufficient to obtain stable aggregated metagene coefficients, as the cophenetic correlation coefficients were not significantly less than *r*_*mf*_=50 or 75 (Supplementary Fig. 3b).

### Evaluating the performance of factorization methods

For LOO-CV-based evaluation, we trained linear support vector machine classifiers by using the factors from (*m*−1) cells and predicted the cell group (Emcn^+^/Gata1^−^/Tbx20^−^, Gata1/Emcn^−^/Gata1^+^/Tbx20^−^ and Emcn^−^/Gata1^−^/Tbx20^+^) of the remaining cell. This procedure was repeated for every single cell and the LOO-CV error was determined as the overall prediction error. Lower LOO-CV error suggested better factorizations on capturing the difference of the three groups of cells. For WSS/TSS ratio-based evaluation, we computed the ratio of WSS and TSS of resulting factors. Lower WSS/TSS ratio suggested that three group of cells were more tightly clustered together on the reduced dimensions.

### Clustering cells into metacells using a SOM

We used SOM to map cells into *P*=225 prototype metacells that were spatially organized on a 15 × 15 2D hexagonal grid^44^. The input space for SOM was the mean metagene expression profiles (metagene coefficients) 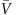 from *r*_*mf*_ repetitive runs of wp-NMF. The R package *kohonen* was used to fit the SOM model with default parameters^66^. We used **W**_*kp*_ to represent scaled expression level of *k*th metagene in metacell *p*, where 
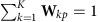
.

### Partitioning SOM using PAM

The SOM were partitioned into multiple segments using PAM algorithm. If the number of desired clusters *C* was specified, the metacells were directly clustered into *C* clusters; otherwise, the SOM would be partitioned into the maximum number of clusters, as long as the size of each metacell cluster was no <15 and every metacell cluster was connected on the SOM (that is, no clusters were divided into two or more isolated regions).

### Constructing a heterogeneous metagene–metacell graph

A transition probability matrix was used to characterize the hierarchical relationships among *P* metacells and between *P* metacells and *K* metagenes. The transition probability matrix was defined as:


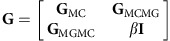


where 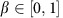 and **I** was a *K* × *K* identity matrix.

As with the metagene entropy for the cells, we defined the metagene entropy of metacell *p* as:


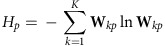


Based on our hypothesis that cells in a progenitor state have higher metagene entropy than cells at the committed state, we initially constructed a *P* × *P* directed metacell graph **G**_MC_ for the hierarchical relationship of metacells. To prioritize committed (progenitor) states, for any metacell *p* on the SOM, the parental metacells were its neighbouring metacells in which metagene entropy was higher (smaller) than *H*_*p*_ and the derivative metacells were the neighbouring metacells where the metagene entropy was lower (higher) than *H*_*p*_.

Thus the similarity between any two metacell *p* and *q* could be computed as:


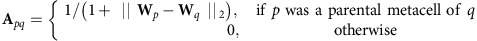


where 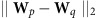 was the Euclidean distance between the metagene coefficients of metacell *p* and *q*.

Finally, the transition probability from metacell *p* to metacell *q*, from metagene *k* to metacell *p* and from metacell *p* to metagene *k* were defined as:


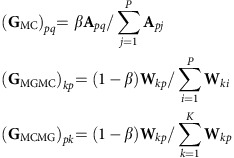


### Prioritizing metacells with respect to cellular states

To prioritize metacells with respect to specified cellular states (committed or progenitor), we utilized a RWR algorithm based on the transition probability matrix **G** (ref. 67). RWR is a method that has been successfully used in numerous item prioritization tasks, such as web searches and characterizing disease-related genes^56^,^68^. The flexibility and robustness of RWR algorithms allowed us to prioritize cells/metacells with defined cellular states. The random walker starts from the vertex representing the metagene(s) and randomly moves to one of its neighbouring metacell or metagene, based on the transition probability described by **G**. Finally, the probability that the random walker reaching a metacell *p* converges to a scaled steady state **u**_*p*_, where 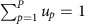, and all the metacell vertices in the graph are ranked by the steady-state probabilities. We used the R package *igraph* to perform the RWR with the default restarting probability 0.85 (ref. 69).

During the random walk, the parameter *β* regulates the probability of staying in the metagene graph. A large *β* encourages the random walker staying in the metacell graph **G**_MC_, resulting in a sharper ranking results, whereas a small *β* encourages the random walker staying in the metacell–metagene graph **G**_MGMC_ and **G**_MCMG_, resulting in a more smoothened ranking. For the results reported in this study, we set *β*=0.85.

### Gene enrichment score

We prioritized genes for a specified cellular state based on the correlation between their expression level in metacells and the steady-state probability **u**. Let **Y**_*np*_ be the expression level of gene *n* in metacell *p*. The enrichment score of gene *n* in prioritized metacells for a specified cellular state was defined as:


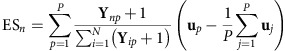


The enrichment score was the sum of steady-state probability (after scaled to mean of zero), weighted by the observed expression level. High enrichment score suggested high correlation between steady-state probability and expression levels.

### Simulating single-cell RNA-seq expression data

We assumed the expected expression level of a gene *n*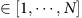 in cell 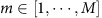, 
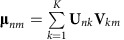
, where **V** was randomly filled with 0 and 1 with probability 0.3 and 0.7, respectively, followed by scaling each column so that 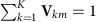 for each *m*, and **U**_*nk*_ was randomly sampled from a Gamma distribution with fixed shape and rate. Let **D**_*nm*_ be a binary indicator matrix of being a dropout event for gene *n* in cell *m*, where the dropout rate is 50%. The observed expression level of gene *n* in cell *m* is **X**_*nm*_, followed a Poisson distribution with mean as **μ**_*nm*_ if **D**_*nm*_=0, otherwise zero. In the experiments, the total number of genes and cells were set to 200 and 50, respectively.

### Data availability

The single-cell RNA-seq data that support the findings of this study have been deposited in NCBI Sequence Read Archive database with the project accession number PRJNA350294. The *dpath* pipeline was implemented as an R package (see Supplementary Software 1). All other relevant data are available from the authors.

## 

## Additional information

**How to cite this article:** Gong, W. *et al*. Dpath software reveals hierarchical haemato-endothelial lineages of Etv2 progenitors based on single-cell transcriptome analysis. *Nat. Commun.* **8,** 14362 doi: 10.1038/ncomms14362 (2017).

**Publisher's note**: Springer Nature remains neutral with regard to jurisdictional claims in published maps and institutional affiliations.

## Supplementary Material

Supplementary Information Supplementary Figures, Supplementary Table, Supplementary Note and Supplementary References

Supplementary Software 1 dpath pipeline implemented as an R package
