# Supplementary material for: Dpath software reveals hierarchical haemato-endothelial lineages of Etv2 progenitors based on single-cell transcriptome analysis
Source: Nat Commun. 2017 Feb 9;8:14362. doi: 10.1038/ncomms14362 (PMC5309826; doi:10.1038/ncomms14362)
Supplement: Supplementary Information — Supplementary Figures, Supplementary Table, Supplementary Note and Supplementary References [file ncomms14362-s1.pdf]

Supplementary Figure 1

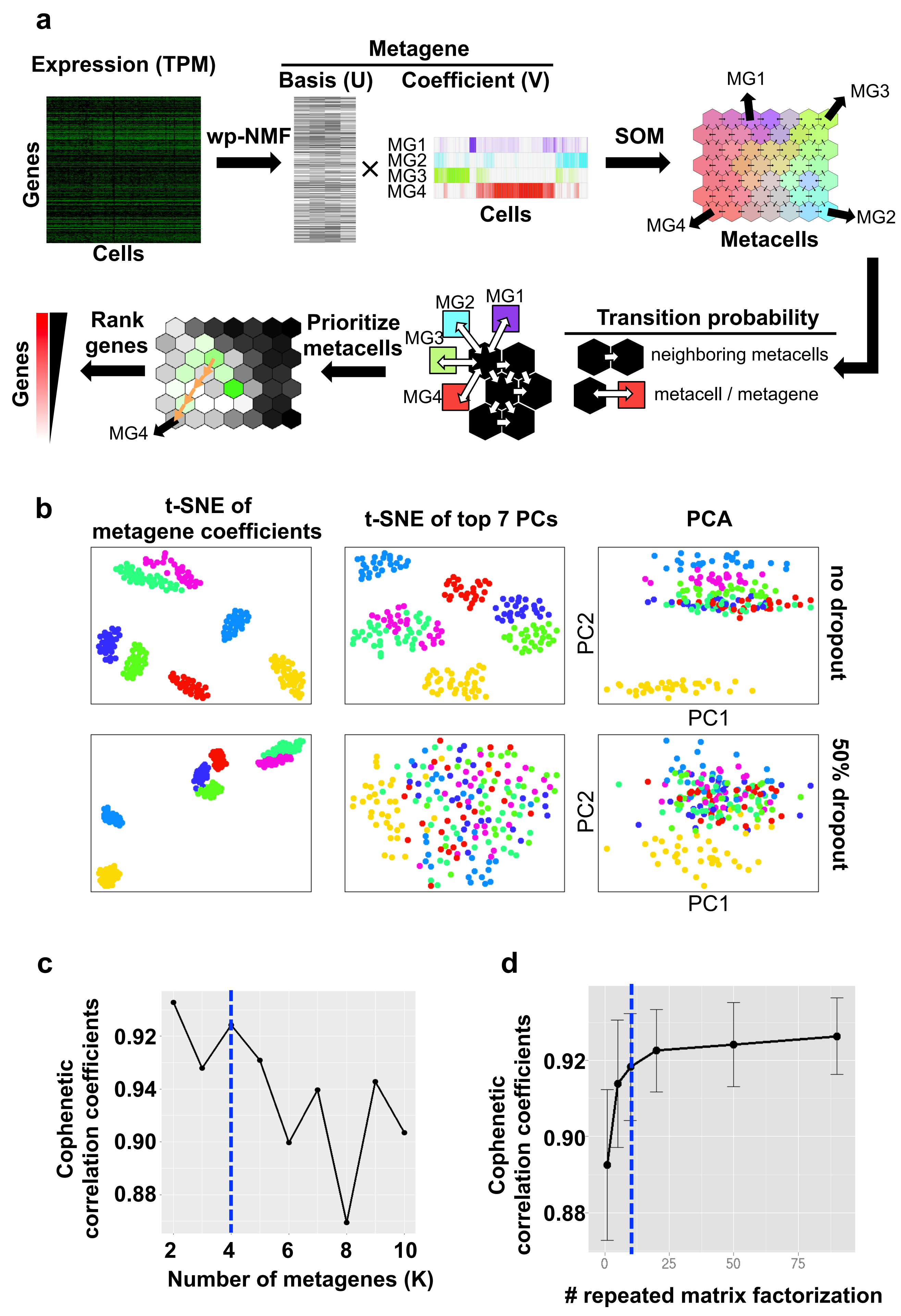

Supplementary Figure 1. Overview of dpath pipeline for analyzing single cell RNA-seq data and the selection of parameters for weighted Poisson non-negative matrix factorization (wp-NMF). (a) A schematic representation of the *dpath* pipeline for analyzing single cell RNA-seq data. The input single cell RNA-seq expression matrix was first decomposed into metagene basis and metagene coefficients using weighted Poisson non-negative matrix factorization (wp-NMF). The resulting metagene coefficients were used to map the cells to a continuous metacell landscape using a self-organizing map (SOM). Differentiated or progenitor cellular states on the SOM were then prioritized based on a random walk with restart algorithm on a heterogeneous metagene-metacell graph. The genes were ranked with respect to each cellular state based on their expression pattern. **(b)** Wp-NMF had superior performance regarding the discovery of hidden cell populations from simulated single cell RNA-seq data with 50% random dropout noise compared to PCA. **(c)** Four metagenes were sufficient to represent the entire components in this Etv2-EYFP single cell RNA-seq dataset. The cophenetic correlation coefficients for different metagene number (K). The blue vertical line indicates the K value where the magnitude of the cophenetic correlation coefficient begins to fall. **(d)** The 20 repetitive runs were sufficient to generate stable factorization results. The scatter plot shows the cophenetic correlation coefficients for different numbers of repeated runs of wp-NMF (r_{mf}). The blue vertical line indicates the r_{mf} number we used in the present study.

Supplementary Figure 2

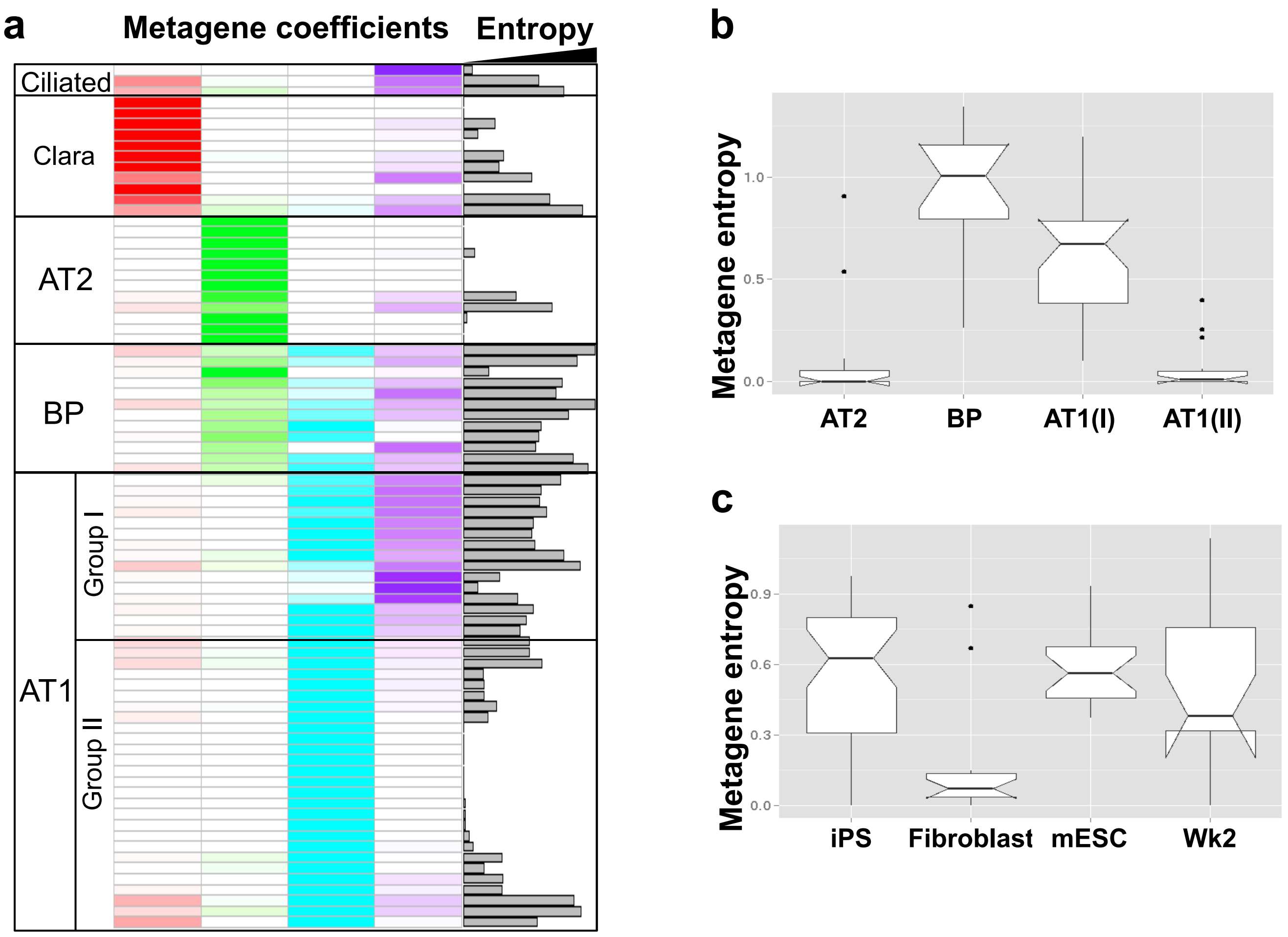

Supplementary Figure 2. Metagene entropy successfully separated progenitor from committed cell populations. (a) The heatmap shows the metagene coefficients for clara, ciliated, flat alveolar type 1 (AT1), surfactant-secreting cuboidal alveolar type 2 (AT2) and bipotential progenitor cells that can give rise to both AT1 and AT2 cells. The color in the left column indicates the metagene expression level in each cell (darker color indicates higher metagene coefficients). The bars in the right column indicate the cell-wise metagene entropy. **(b)** The box plot shows the metagene entropy for the two groups of AT1 cells, AT2 cells and BP cells from Treutlein et al., 2014. Consistent with known information, the results demonstrated that the bipotential (BP) cells that can give rise to both flat alveolar type 1 (AT1) cells and surfactant-secreting cuboidal alveolar type 2 (AT2) cells have significantly higher metagene entropy than committed AT1 or AT2 cells, verifying the prediction power of this method (Wilcoxon rank sum test, *p*-value=1.4×10^{-6}). In addition, our method further identified two distinct populations of AT1 cells (Group1 and Group2), which were not identified using conventional methods. **(c)** The boxplot shows the metagene entropy for iPS, fibroblast, mouse ESC (mESC) and week 2 cells from Kim et al., 2015. The iPS cells had significantly higher metagene entropy than the fibroblast cell population (Wilcoxon rank sum test, *p*-value=2.4×10^{-10}).

Supplementary Figure 3

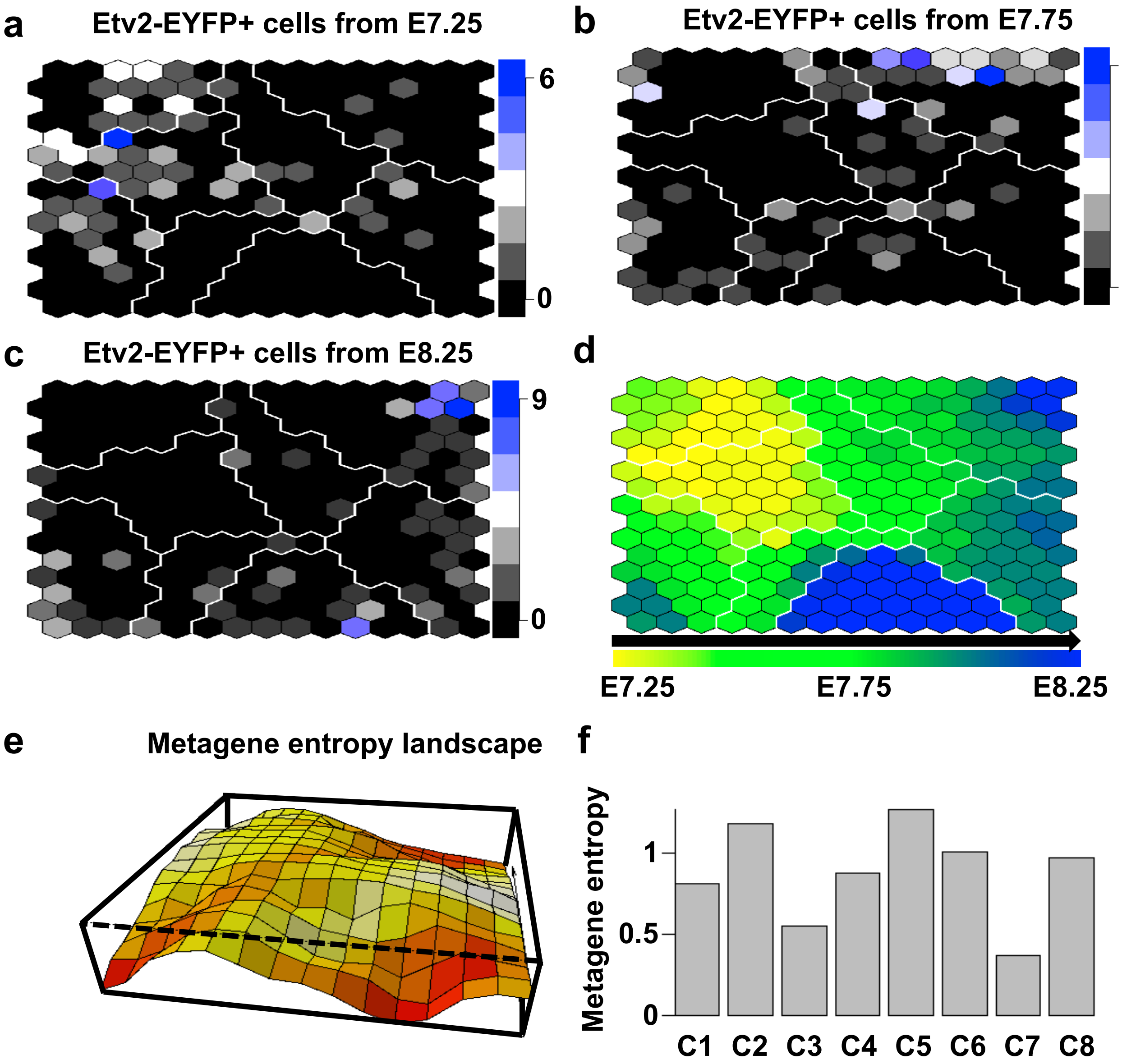

Supplementary Figure 3. The Etv2-EYFP+ cells from E7.25, E7.75 and E8.25 had distinct distributions on the metagene entropy landscape. **(a-c)** The distribution of the number of metacells from **(a)** E7.25, **(b)** E7.75 and **(c)** E8.25 was illustrated on the SOM. Dark blue indicates 100% of cells within the metacell are from the respective time point and black indicates 0% of the cells are derived from the respective time point. **(d)** The distribution of the cells' temporal sources was illustrated on the SOM. The metacell with yellow, green and blue color indicated that the majority of the cells mapped to this metacell came from E7.25, E7.75 and E8.25 embryos, respectively. **(e)** The 3D contour plot shows the metagene entropy landscape on the SOM. The metacell landscape represented the lineage relationships reminiscent of the branching valleys of the Waddington's epigenetic landscape. **(f)** The baraplot shows the average metagene entropy of cells from each of the eight clusters.

Supplementary Figure 4

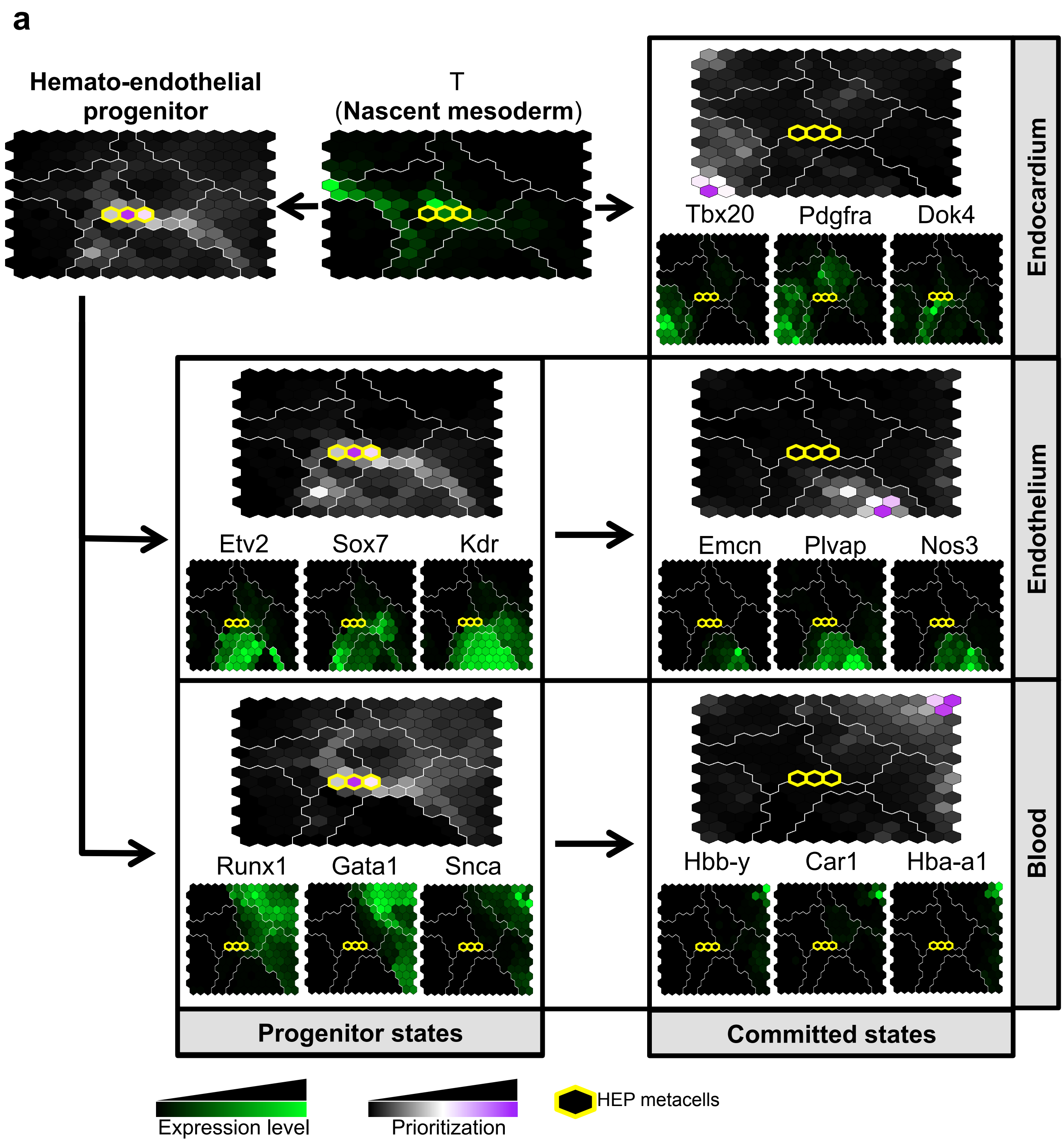

Supplementary Figure 4

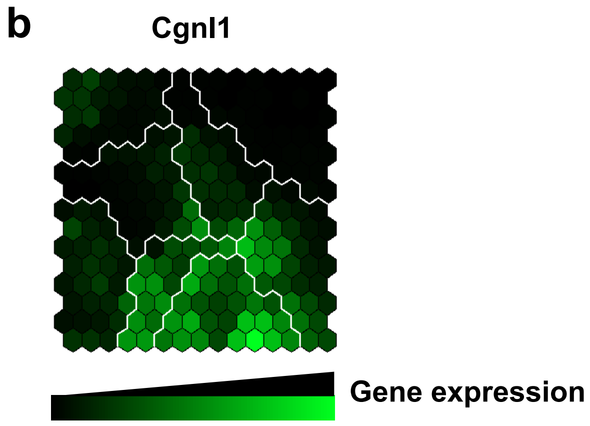

Supplementary Figure 4. The SOM allows the visualization of cellular (committed or progenitor) states in the process of differentiation from mesodermal progenitors to endocardial, endothelial and hematopoietic lineages. (a) The SOMs with black and purple color represent the prioritized metacells for different cellular states, where purple and black colors indicate high and low prioritization scores, respectively. The SOMs with black and green colors show the expression pattern of representative genes for each cellular state, where the green and black colors indicate high and low expression levels, respectively. The top three prioritized progenitor metacells are highlighted by yellow lines. Metacells with the highest T expression levels are marked with an asterisk. HEP: high entropy progenitors. **(b)** The expression pattern of additional selected genes were illustrated on the SOM. Green: high expression. Black: low expression.

Supplementary Figure 5

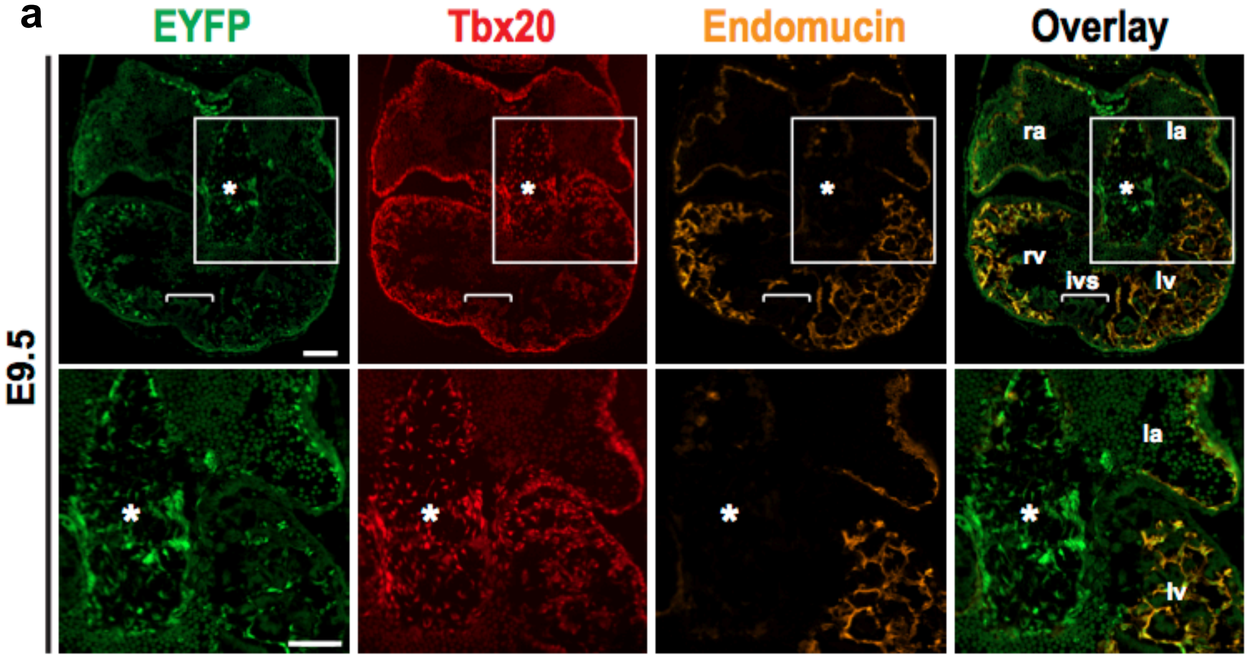

Supplementary Figure 5. Immunohistochemical analysis of Etv2-EYFP transgenic hearts showed Etv2 and Tbx20 co-expression in the E9.5 endocardial cushion of the developing mouse. (a) Fluorescent images were pseudo-colored after photographing in black and white (a: common atrium, cc: cardiac crescent, ec: endocardium, ivs: intraventricular septum, la: left atrium, lv: left ventricle, nt: neural tube, oft: outflow tract, ra: right atrium, rv: left ventricle). The asterisk and bracket indicate endocardial cushion and the developing intraventricular septum, respectively. Boxed areas were enlarged in the images below. Scale bars indicate 100 μm.

Supplementary Figure 6

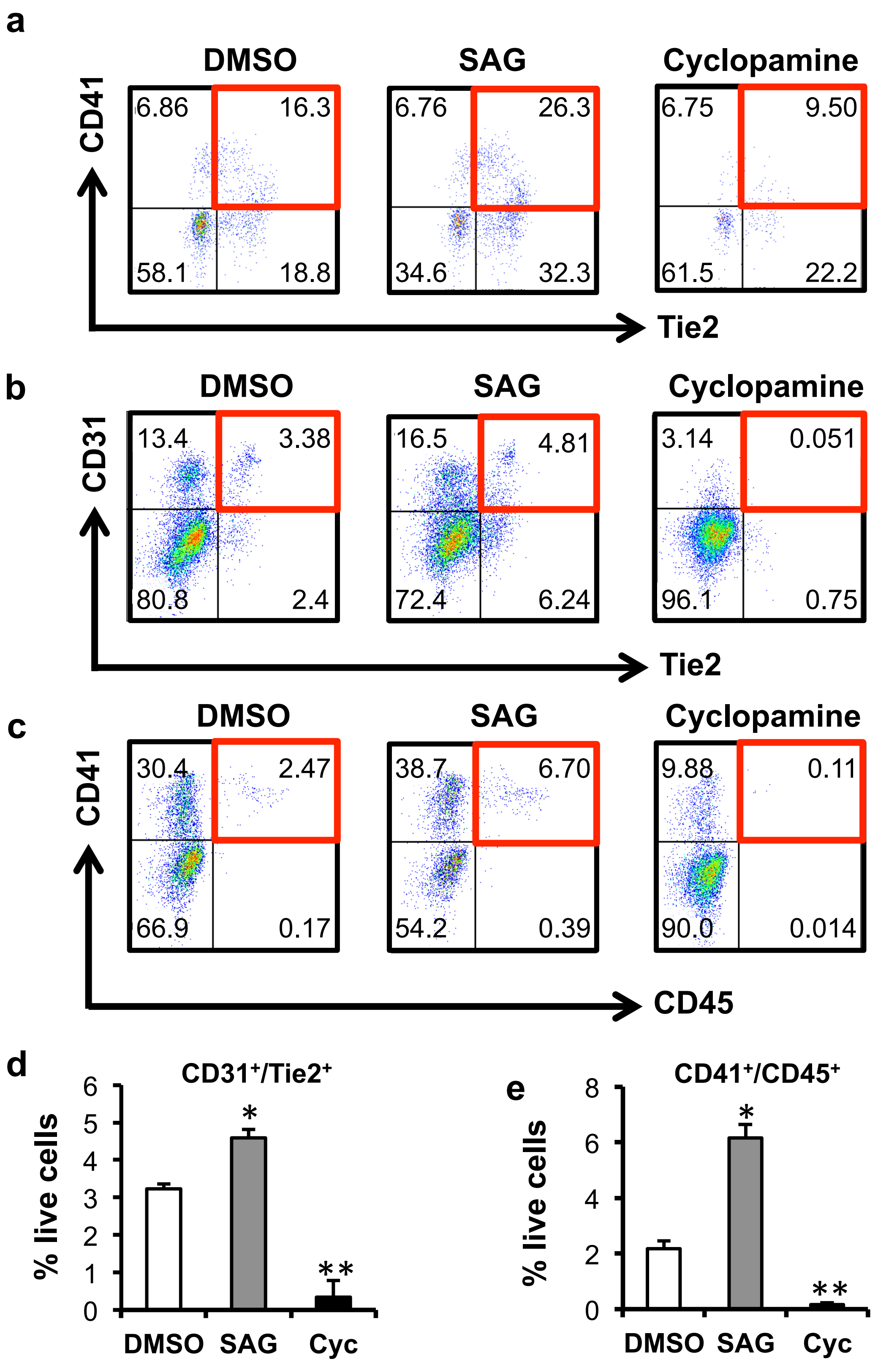

Supplementary Figure 6. The Sonic Hedgehog signaling (SHH) pathway regulates the differentiation of hemato-endothelial lineages. FACS profiles indicates that sonic hedgehog agonist (SAG) (or cyclopamine) significantly promote (or suppress) endothelial and hematopoietic progenitors ($CD41^+/Tie2^+$), compared with DMSO control (*: p-value < 0.05). **(b-e)** FACS profiles and quantification of endothelial ($CD31^+/Tie2^+$, panel **b** and **d**) and hematopoietic ($CD41^+/CD45^+$, panel **c** and **e**) indicates that sonic hedgehog agonist (SAG) (or cyclopamine) significantly promote (or suppress) endothelial and hematopoietic lineages, compared with DMSO control (*: $0.01 \leq p$ value < 0.05; **: $0.01 \leq p$ value < 0.01).

Supplementary Figure 7

a. committed hematopoietic (MG2)

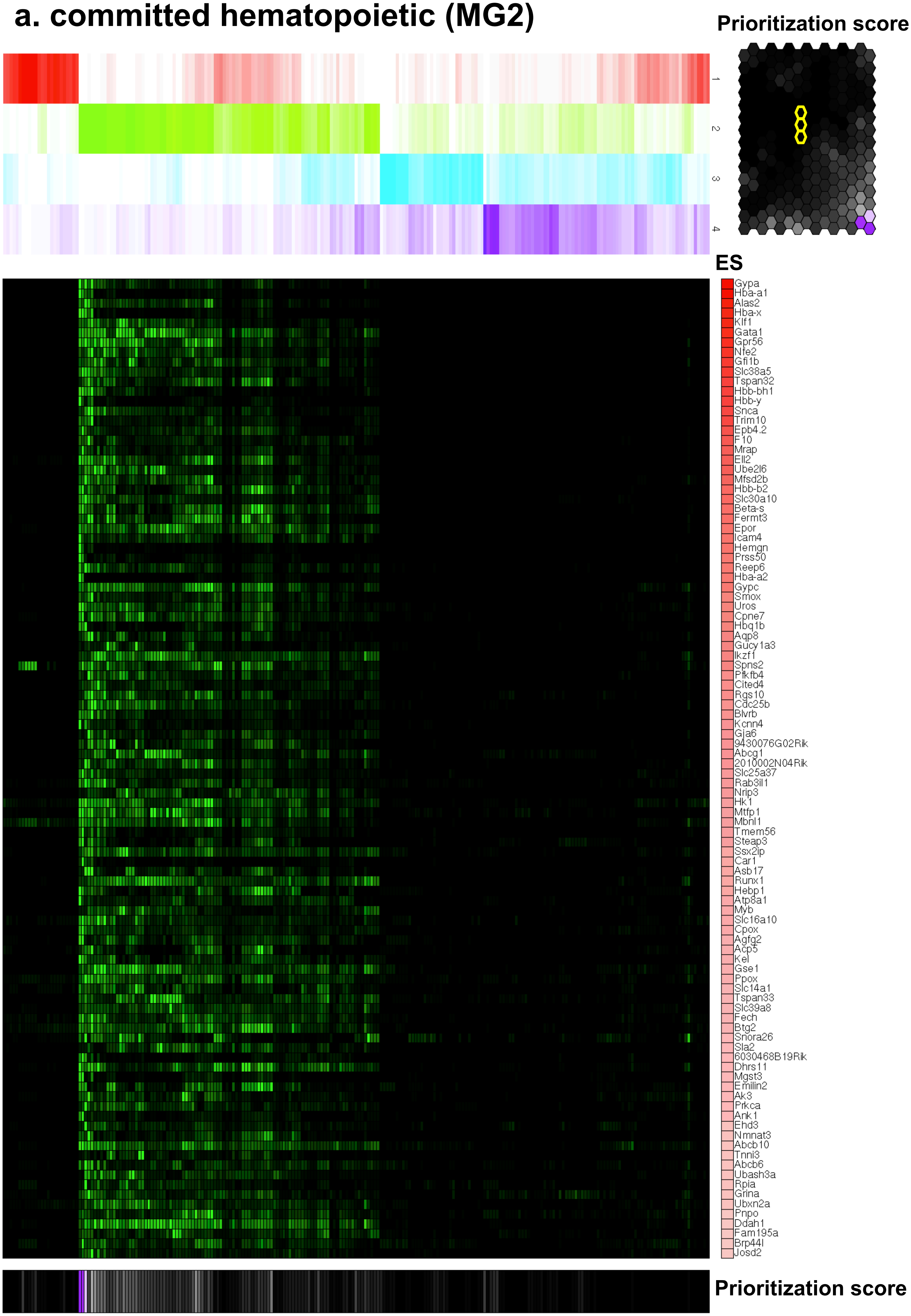

Supplementary Figure 7

b. committed endocardial (MG3)

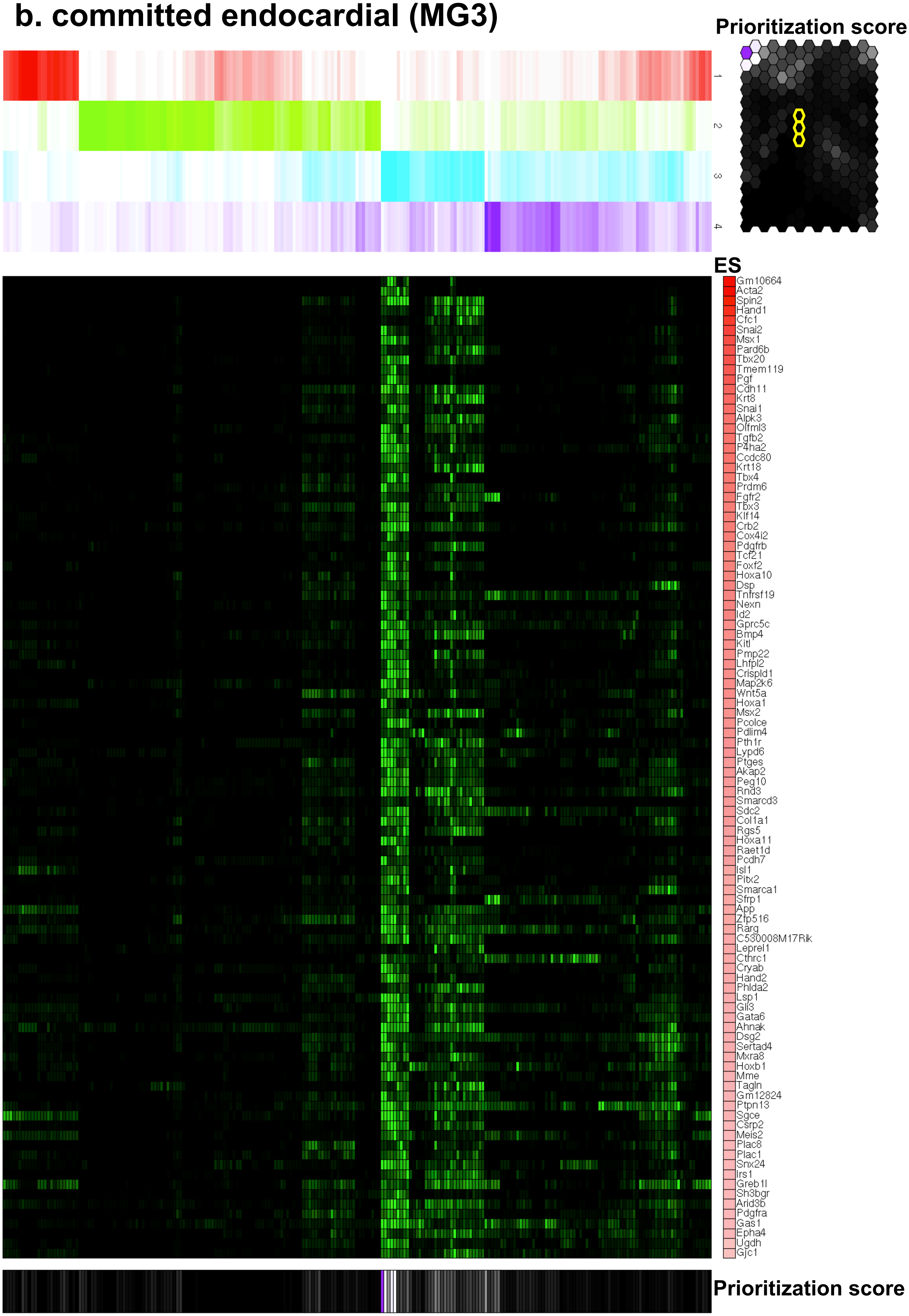

Supplementary Figure 7

c. committed endothelial (MG1)

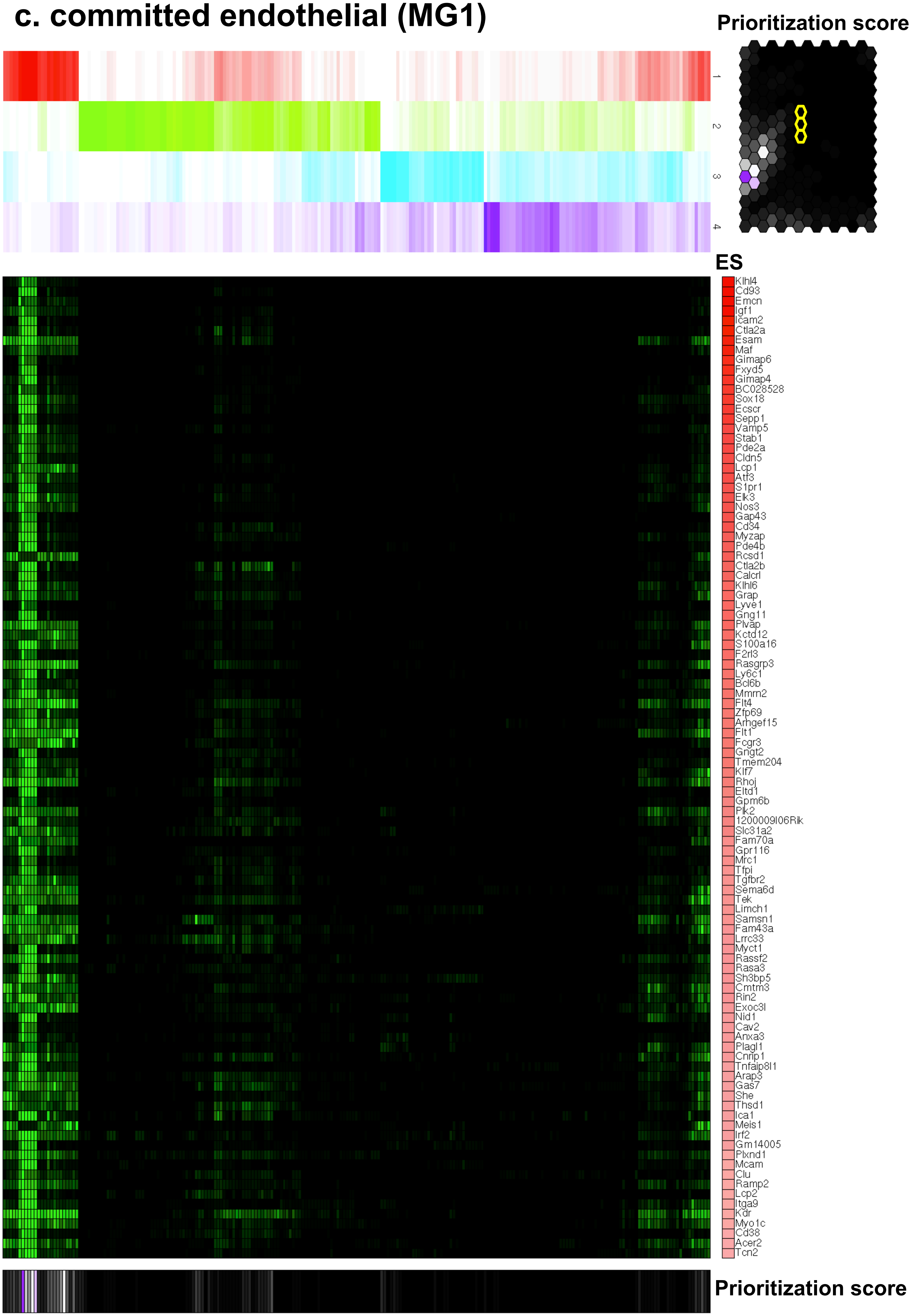

Supplementary Figure 7

d. hematopoietic progenitors

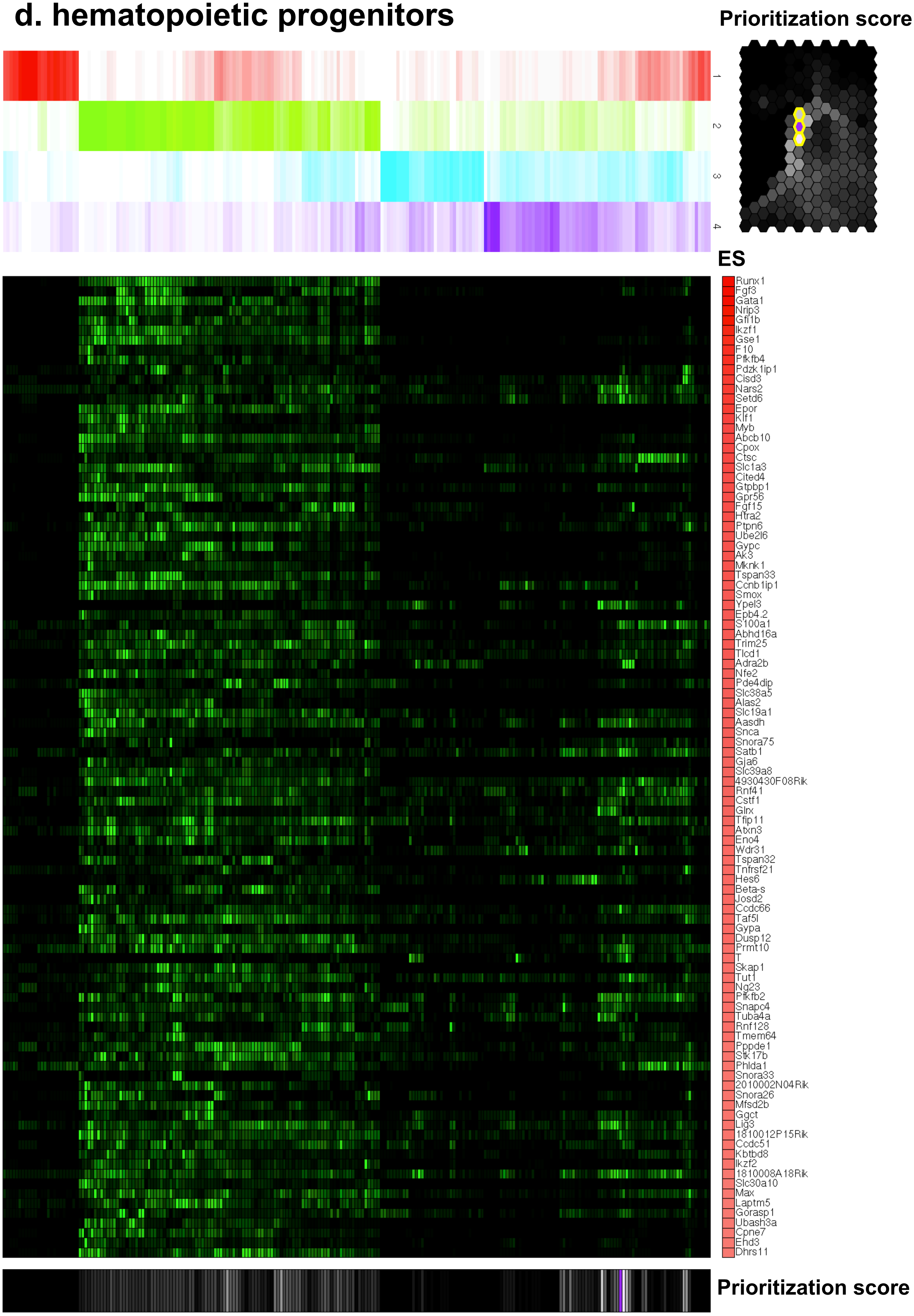

Supplementary Figure 7

e. endothelial progenitors

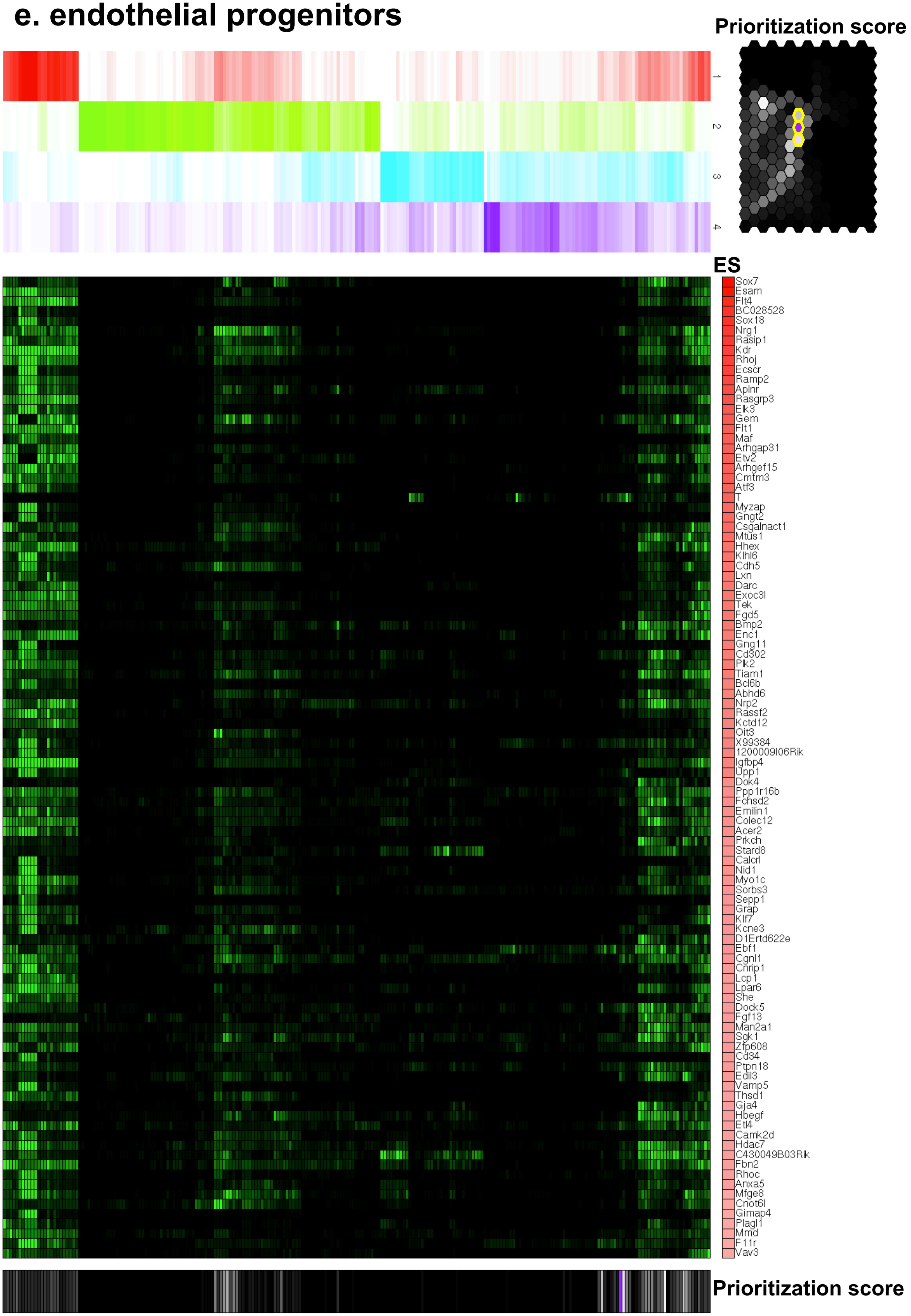

Supplementary Figure 7

f. multi-potent progenitors for hematopoietic and endothelial lineages

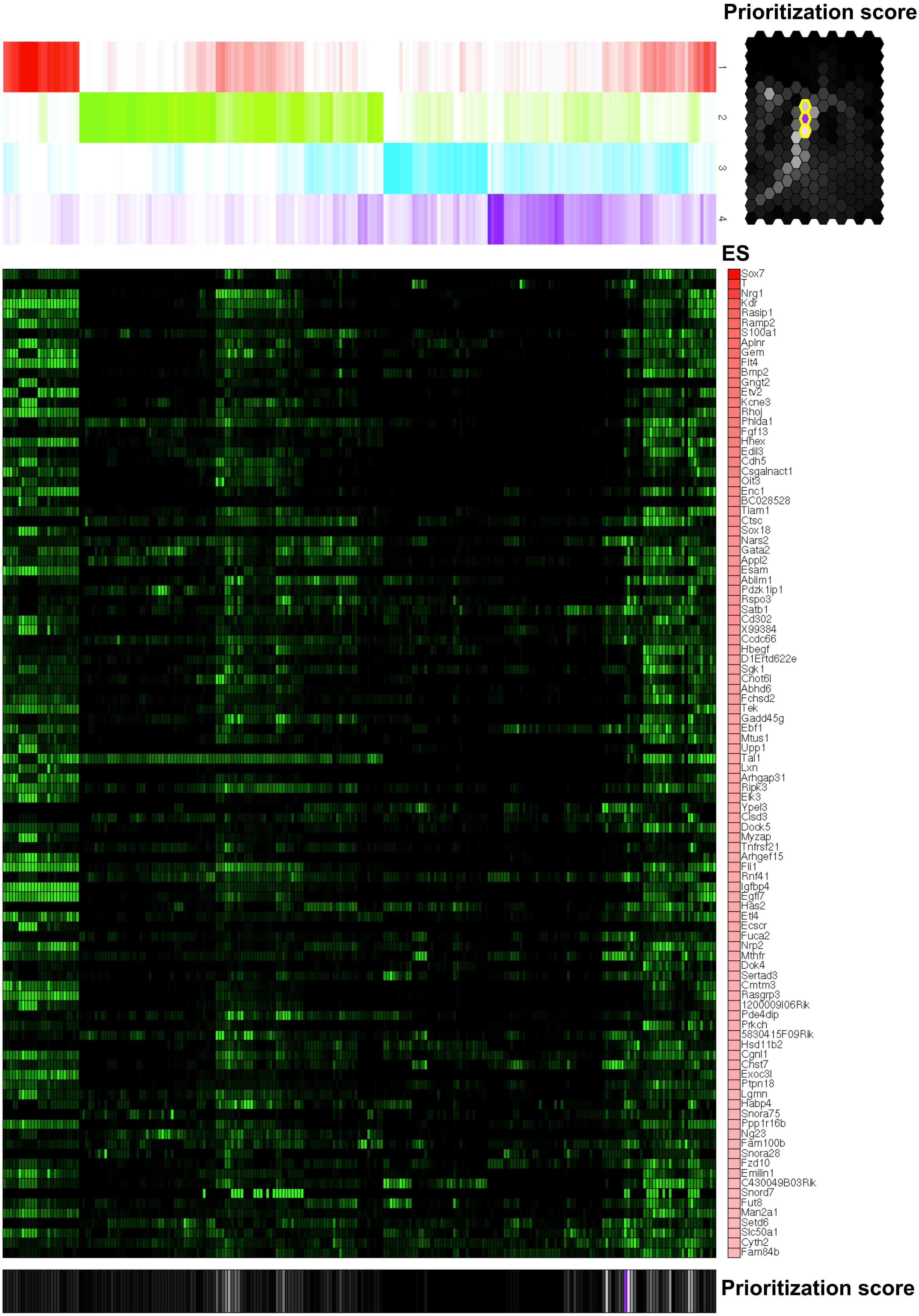

Supplementary Figure 7. dpath prioritized metacells with specific cellular states and specifically expressed genes. Each sub figure shows the top 100 ranking genes that have an expression pattern correlated with the prioritization score with respect to **(a)** committed hematopoietic (MG2) **(b)** committed endocardial (MG3), **(c)** committed endothelial (MG1), **(d)** hematopoietic progenitors, **(e)** endothelial progenitors and **(f)** multi-potent progenitors for hematopoietic and endothelial lineages. In each plot, the top right panel shows the prioritization score on the SOM, where the purple and black colors indicate high and low prioritization scores, respectively. The top left panel shows the metagene expression profile in each metacell, where the intensity of the green color indicates the expression intensity. The middle panel shows the gene expression pattern in metacells.

Supplementary Figure 8

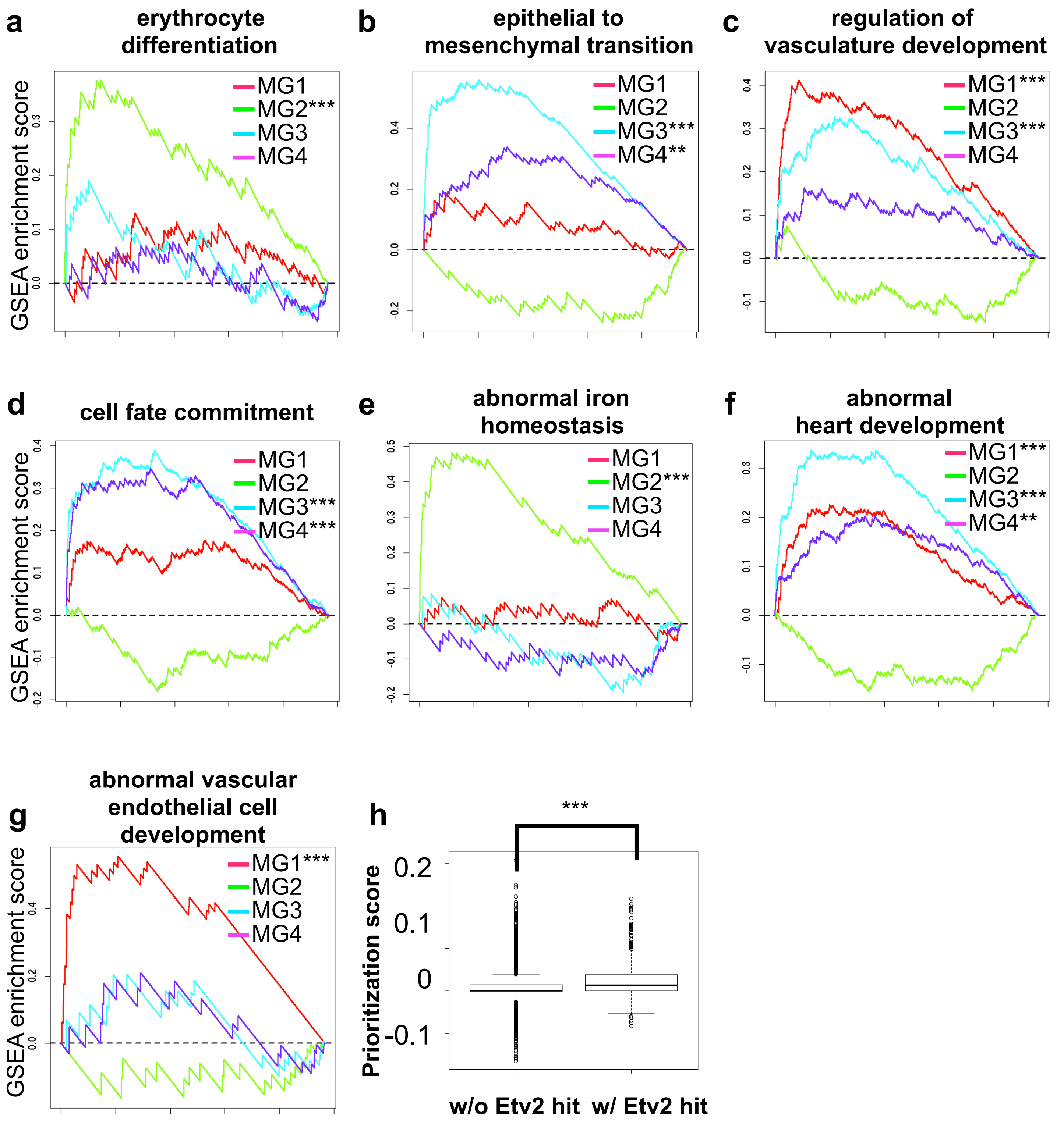

Supplementary Figure 8. Genes ranked by dpath with respect to specific cellular states were significantly associated with known biological functions. **(a-g)** Gene set enrichment analysis (GSEA) shows that genes ranked by the correlation between their expression pattern and prioritization score with respect to the committed states of four metagenes were associated with distinct biological functions. **(a)** Erythrocyte differentiation (GO:0030218). **(b)** Epithelial to mesenchymal transition (GO:0001837). **(c)** Regulation of vasculature development (GO:1904018). **(d)** Cell fate commitment (GO:0045165). **(e)** Abnormal iron homeostasis (MP:0005637). **(f)** Abnormal heart development (MP:0000267). **(g)** Abnormal vascular endothelial cell development (MP:0003542). **(h)** The prioritization scores of genes with Etv2-ChIP-seq hits (reported by Liu et al.) were significantly higher than genes without the ChIP-seq hits. *: $0.01 \leq p$ value < 0.05; **: $0.001 \leq p$ value < 0.01; $***$: p value < 0.001.

Supplementary Figure 9

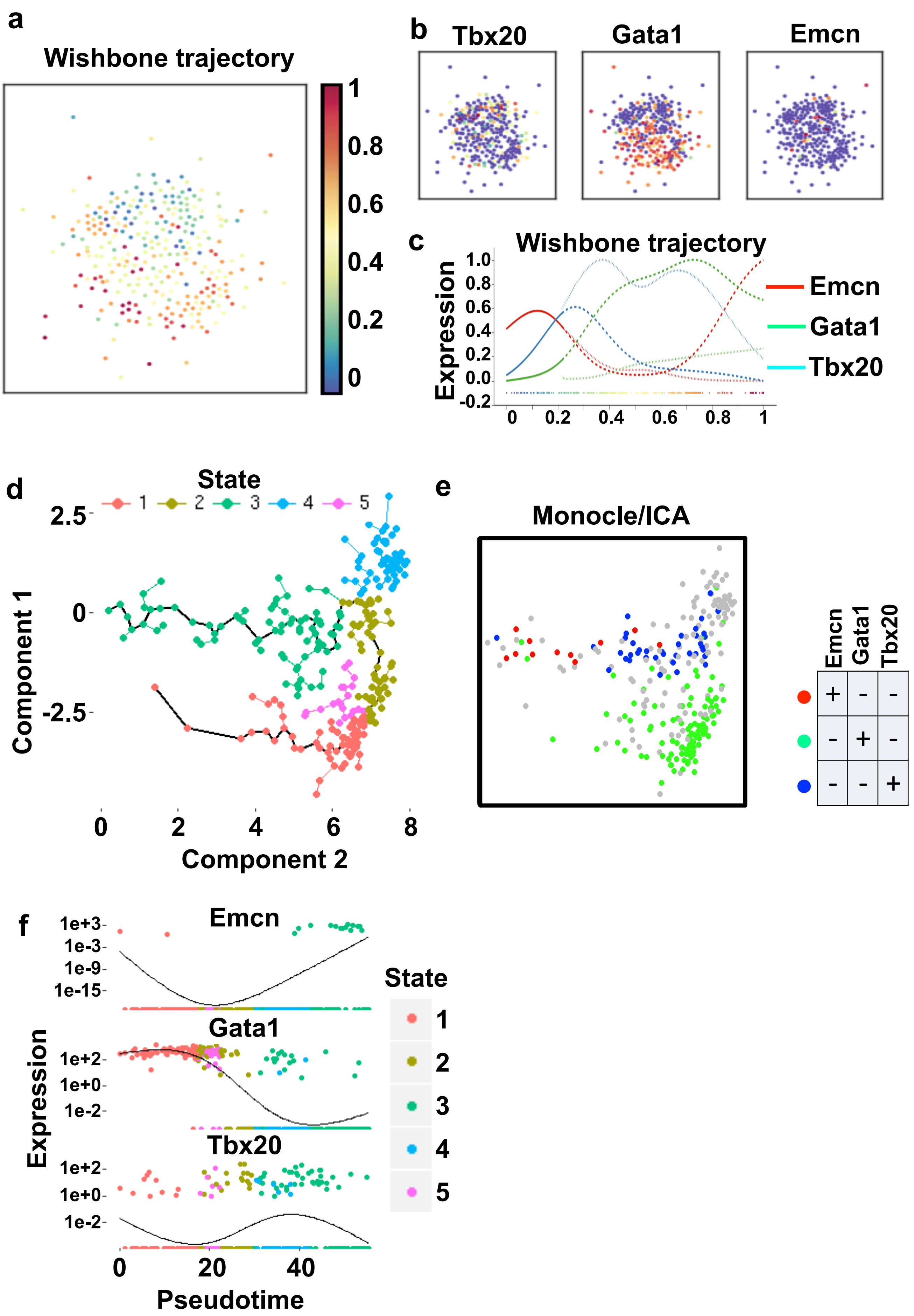

Supplementary Figure 9

g

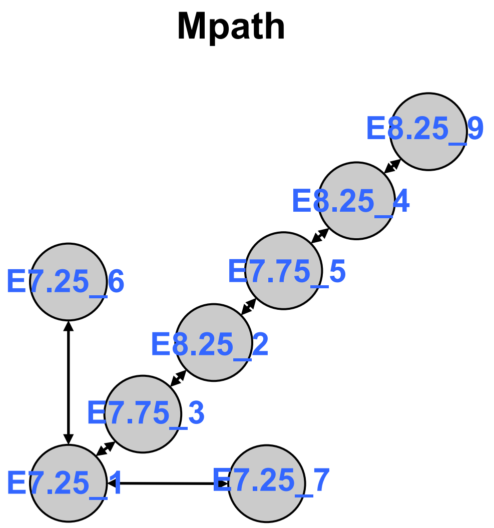

h

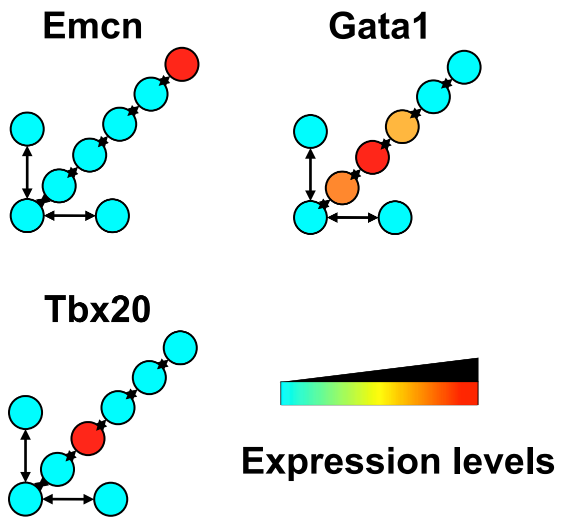

Supplementary Figure 9. dpath had superior performance than Wishbone, Monocle and Mpath for revealing developmental trajectories from Etv2-EYFP+ single cell data. **(a)** The scatter plot shows the developmental trajectories inferred by Wishbone. **(b-c)** The scatter plot shows the expression pattern of Emcn, Gata1 and Tbx20 along the Wishbone trajectories. **(d)** The scatter plot shows the developmental trajectories inferred by Monocle. **(e)** The scatter plot shows the distribution of $Emcn^{+}/Gata1^{-}/Tbx20^{-}$, $Emcn^{-}/Gata1^{+}/Tbx20^{-}$ and $Emcn^{-}/Gata1^{-}/Tbx20^{+}$ cells on the two dimensional space represented by Monocle. **(f)** The expression of Emcn, Gata1 and Tbx20 along the Monocle's pseudotime. **(g)** The undirected graph illustrates the branching trajectory determined by Mpath. **(h)** The expression of Emcn, Gata1 and Tbx20 was illustrated on Mpath's branching trajectory.

Supplementary Table 1. Number of embryos, average number of Etv2-EYFP+ cells per embryo, number of captured Etv2-EYFP+ cells and number of cells for retained after quality filtering for each developmental stage (E7.25, E7.75 and E8.25).

	# embryos	Average # Etv2-EYFP+ cells per embryo	# captured Etv2-EYFP+ cells	# cells passed QC
E7.25	31	350	86	83
E7.75	10	1500	100	99
E8.25	5	6500	100	99

Supplementary Note 1

The C1 cell population having an $Etv2^+/Tbx20^+/Emcn^-$ signature

The clustering of 281 Etv2-$EYFP^+$ single cells by portioning SOM using PAM algorithm yielded eight major cell groups with distinct metagene signatures (Figure 3d and 3e)[1]. Among the eight cell groups, C1 harbored the metagenes (MG3 and MG4) for cardiac and mesodermal progenitors. We compared the metagene signature of the C2 population, which harbored the metagenes for endothelial, cardiac and mesodermal progenitors (MG1, MG3 and MG4) and we predicted that the C2 population was the progenitors of the C1 population. The gene profile analysis revealed that the general gene expression change is C2 (Etv2-$EYFP^+$, $Cardiac^+$, $Endothelial^+$) → C1 (Etv2-$EYFP^+$, $Cardiac^+$, $Endothelial^-$). This transition is similar to the endothelial-mesenchymal transition (EMT) involved in the generation of cardiac cushion from the endocardium[2]. However, this shift was already detected between E7.75 and E8.25 in our analysis, which was approximately a half day earlier than the appearance of the morphologically distinct cardiac cushion[3]. To define the timing of the appearance of the C1 cells, we morphologically examined the heart loop at E7.75, E8.5 and E9.5. The combinations of markers (EYFP driven by the Etv2 promoter, Tbx20, and endomucin) showed a clear distinction between the C1 and C2 population. At E7.75, EYFP cells were restricted to the developing dorsal aortae (Figure 4b, large arrowheads) and isolated angioblasts in the lateral plate mesoderm and in the $Tbx20^{dim}$ cardiac crescent (Supplementary Figure 4b, small arrows). These EYFP positive cells co-localized with endomucin, validating the C1 and C3 profiles. At E8.5, the endocardium expressed EYFP, Tbx20, and endomucin (the C2 signature), however, a distinct population that expresses EYFP (dim) and Tbx20, but not endomucin (the C1 signature) appeared on the inner curvature of ventricular and atrial walls (small arrows). These cells were distinct from the endocardial population, which has not fused with the myocardium at this stage. At E9.5, the cells with the C1 profile were localized to the cardiac cushion, which are the precursors of valves (see Supplementary Figure 5a, star). EYFP was down-regulated in many endocardial cells, however all remaining EYFP positive cells were also positive for endomucin, showing the C2 signature. The ventricular cushion, the precursor of interventricular septum was negative for Etv2 and endomucin, indicating that formation of interventricular septum is regulated by a distinct molecular pathway (Figure 4b, bracket). Collectively, these data suggest that the C1 population are progenitors of the cardiac cushion that originate form endocardium, and the molecular transition (i.e. changes in gene expression profile) occurs as early as E8.25. The first appearance of immunohistochemically detectable C1 population was E8.5.

References

1. Van der Laan, M. J. & Pollard, K. S. A new algorithm for hybrid hierarchical

clustering with visualization and the bootstrap. *Journal of Statistical Planning and Inference* **117,** 275–303 (2003).
2. Gise, von, A. & Pu, W. T. Endocardial and epicardial epithelial to mesenchymal transitions in heart development and disease. *Circ. Res.* **110,** 1628–1645 (2012).
3. Snarr, B. S., Kern, C. B. & Wessels, A. Origin and fate of cardiac mesenchyme. *Dev. Dyn.* **237,** 2804–2819 (2008).
